# Novel Cyanopyrimidine Derivatives as Potential Anticancer Agents

**DOI:** 10.3390/molecules30071453

**Published:** 2025-03-25

**Authors:** Rania H. Abd El-Hameed, Omnia Aly, Mariem E. Mohamed, Amal F. Gharib, Mosaad S. Mohamed, Ashraf Ali, Zainab M. Khoder, Heba Taha, Samar S. Fatahala

**Affiliations:** 1 Pharmaceutical Organic Chemistry Department, Faculty of Pharmacy, Helwan University, Cairo 11795, Egypt; zeiadomar@yahoo.com (R.H.A.E.-H.); mariem.elsaeed@pharm.helwan.edu.eg (M.E.M.); mosaad_abdlallah@pharm.helwan.edu.eg (M.S.M.); zkhoder@buffalo.edu (Z.M.K.); 2 Medical Biochemistry Department, National Research Centre, Dokki 12622, Egypt; oe.el-sayed@nrc.sci.eg; 3 Department of Clinical Laboratory Sciences, College of Applied Medical Sciences, Taif University, Taif 21944, Saudi Arabia; dr.amal.f.gharib@gmail.com; 4 Department of Reproductive Health Research and Family Planning, National Research Centre, Dokki 12622, Egypt; doctorashraf1981@yahoo.com; 5 Department of Chemistry, The State University of New York, Buffalo, NY 14260, USA; 6 Biochemistry and Molecular Biology Department, Faculty of Pharmacy, Helwan University, Cairo 11795, Egypt; heba.taha@pharm.helwan.edu.eg

**Keywords:** breast cancer, ovarian cancer, SKOV3, Mcl-1, Bcl-2, docking, cell cycle

## Abstract

The Bcl-2 family’s anti-apoptotic proteins, particularly Mcl-1, offer a viable avenue for cancer treatment since cancer cells can undergo apoptosis when their selective suppression occurs. Mcl-1 is essential for controlling the advancement of the cell cycle, as well as apoptosis. There is a constant clinical need for more potent treatments for breast and ovarian malignancies, even with advancements in the discovery of anticancer drugs. By synthesizing cyanopyrimidine derivatives that demonstrate both dual inhibitory activity against Mcl-1 and Bcl-2, and successful cell cycle arrest, our research seeks to contribute to the development of innovative therapeutic medicines. We created a number of new 6-substituted cyanopyrimidines and tested their anticancer effects on SKOV-3 and MCF-7 cell lines as well as apoptosis and cell cycle arrest assays.

## 1. Introduction

Breast cancer (BC), a non-communicable disease, is one of the most prevalent forms of cancer in women. In 2020 there were more than 2 million new cases, and over the years its rate increased more and more. It is also considered the fifth cause of cancer-related deaths. BC accounts for 14% of deaths in Egypt after cardiovascular disorders (46% percent). According to WHO in 2021, Egypt has a comprehensive strategy in place to prevent and control cancer. Numerous interventions have been undertaken over the years to enhance cancer prevention, screening, treatment and early diagnosis in Egypt, all of which contribute to the country’s improvement of health outcomes [1,2]. When comparing Egypt with developed countries, the mortality/incidence rate ratio for BC is nearly double (41% vs. 23%) [3,4].

It is noteworthy that 5–10% of patients with breast cancer have a genetic predisposition to other types of cancer. According to the Houston Methodist Main Site, nowadays it is crucial to take into account whether there is a present connection between female ovarian cancer and breast cancer when discussing this deadly sickness [5]. Concentrating on both kinds reveals that a network of genetic and biological connections are unite them. The term “hereditary breast and ovarian cancer” (HBOC) syndrome is frequently used to describe this genetic relationship between both BC and Ovarian cancer [6]. It is now advised that women with ovarian cancer undergo genetic testing in order to assist them to control their risk of BC.

An elevated risk of breast cancer, ovarian cancer, as well as other cancers like prostatic, pancreatic, and melanoma, is a characteristic of BRCA1/2-associated HBOC. BRCA1 and BRCA2 are the most frequently found genes that increase the risk of breast cancer [7]. According to the mechanisms of action of BRCA1/2 in breast oncogenesis, BRCA1/2 is involved in apoptosis regulation [8]. BRCA1/2, as caretaker tumor suppressor genes, promote the repairing of DNA double-strand breaks, using homologous recombination repair (HRR). Furthermore, they regulate the dynamics of centrosomes, and they maintain the temporal and spatial stability of the genome throughout the cell cycle. BRCA1 has multiple roles, including initiating breast and ovarian cancer, in addition to its involvement in repairing DNA damage [9]. In light of this, any mutation or loss of the BRCA gene affects the DNA repair process, which causes damaged DNA to accumulate and raises the risk of ovarian and breast cancer. Triple-negative breast cancer (TNBC) is more common in those with BRCA mutations, indicating a potential connection between the hormone receptor status and BRCA mutation [10].

**Figure 1:**
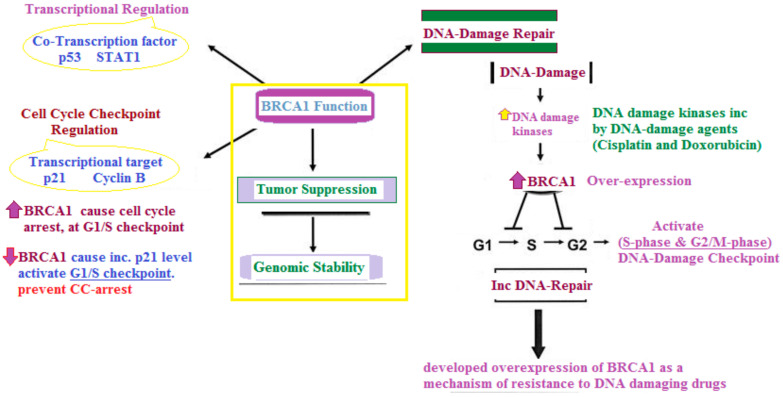
Main role of BRCA-1 in both cell cycle regulation and chemotherapy drug resistance [13].

Another effect is now under consideration due to the loss/mutation of the BRCA1/2 gene in BC/OC patients; the BRCA1 gene, encoding a nuclear protein on respond to DNA damage, participates in cellular pathways responsible for DNA repairs and cell cycle regulation [11]. Given that the majority of chemotherapeutic agents operate by directly or indirectly inflicting damage on DNA, the activity of BRCA1 following chemotherapy-induced DNA damage and its potential as a biomarker for response to these agents has been the focus of numerous investigations, as depicted in Figure 1 [12].

**Figure 2:**
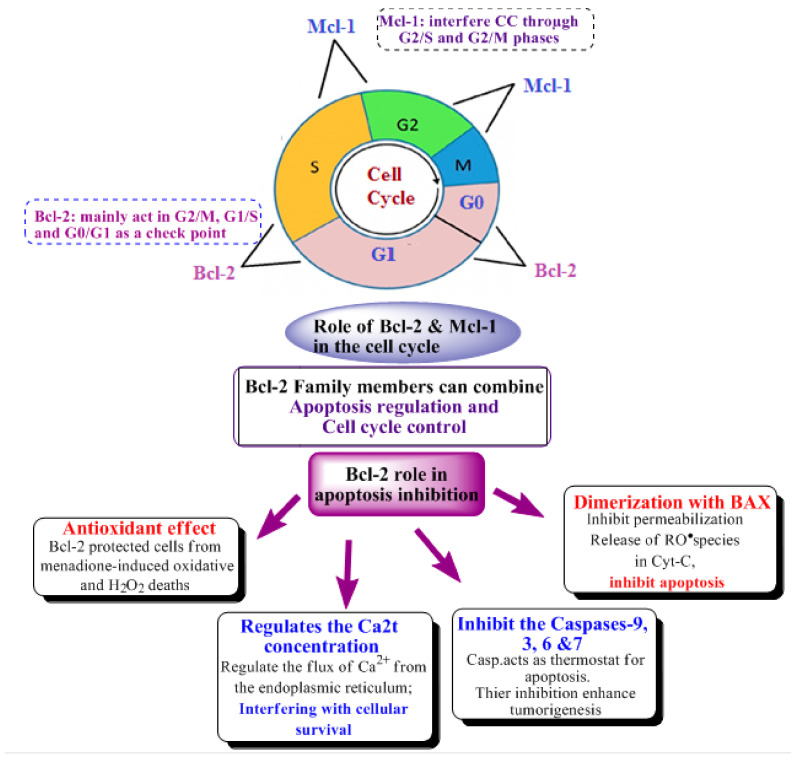
Role of Bcl-2 family members in apoptosis induction, as well as its anti-cancer effect through apoptosis inhibition [15] and cell cycle control [16,17,18].

Concern was raised about the expression of the pro-apoptotic p53 gene and the anti-apoptotic B-cell lymphoma 2 (Bcl-2/Mcl-1) gene in BRCA-associated breast carcinomas [14], as revealed in Figure 2.

The BC has now been classified into: HER2-enriched or basal-like, Luminal A, and Luminal B [4]. Each anti-BC drug has its own mechanism according to the subtype it can interact with [19]—some can interfere with the structure of DNA (such as Gemcitabine), block proteins in the cell (such as Palbociclib), or induce apoptosis (such as Bortezomib) [20]. Others can combine apoptosis regulation [21].

BC occurrence was not only related to the mutation of the oncogenes, but was also related to apoptosis inhibition. Accordingly, the Bcl-2 Protein involved in cell apoptosis is highly related to the proliferation of BC and its occurrence. The Bcl-2 family is classified into three groups which are anti-apoptotic, pro-apoptotic initiators and pro-apoptotic effectors, according to the type of homology motifs “BH regions” they share, for example the anti-apoptotic group which consists of Bcl-2, Bcl-B, Bcl-x_L_, Bcl-w, Al, Mcl-1 share three BH regions [22,23,24,25,26,27], as revealed in Figure 2.

Interference with the cell cycle is controlled, it is also one of the main anti-cancer mechanisms performed by many different proteins’ tyrosine kinases, and it regulates cell functions such as differentiation, proliferation and anti-apoptotic signaling. Recently, pyrimidines bearing compounds act mainly by interfering with the kinases, and their main mechanism inhibits CDKs, as well as myeloid cell leukemia 1 (Mcl-1), proving that the drugs can act by more than a mechanism and showing Mcl-1’s role in cell cycle regulation [28] (as shown in Figure 2; Bcl-2’s and Mcl-1’s effect on the cell cycle) [21,29,30,31].

DNA damage response (DDR) is a mechanism frequently utilized by cancer cells to sustain survival while allowing for genetic mutations [32,33]. The pro-apoptotic Mcl-1 encourages DNA damage response (DDR) not only as an apoptotic switch but also as a crucial factor in DDR activation [14,34.] Mcl-1 was overexpressed in response to DNA damage caused by radiation and/or therapeutic alkylating drugs [34,35]. Mcl-1 mainly promotes the activation of different protein kinase responsible for interfering with the cell cycle responsible for DNA repair through homologous recombination (HR) [14,36]. Ultimately, this could provide a novel area for combination therapy with Mcl-1 inhibition and DNA-damaging agents as a novel therapeutic strategy in cancer [14,27].

## 2. Results and Discussion

### 2.1. Chemistry

**Figure 3:**
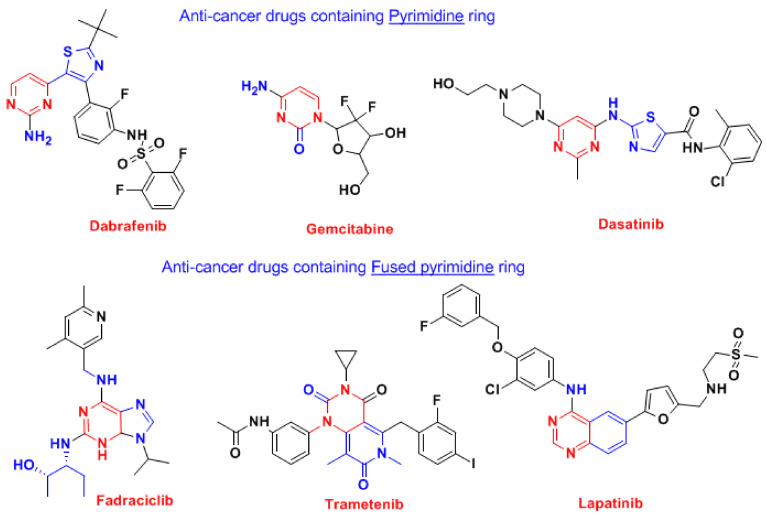
FDA-approved anti-cancer drugs containing pyrimidines [37,38,39,40].

Given the significant impact of pyrimidine and fused pyrimidine derivatives as promising FDA-approved anti-cancer agents, their essential function in cell cycle arrest, and the necessity for selective medicines with lower resistance levels, our objective is to create novel 6-substituted cyanopyrimidines exhibiting effective cell cycle arrest and Mcl-1/Bcl-2 inhibitory activity by modifying Mcl-1 and Bcl-2 candidates, as revealed in Figure 3.

**Figure 4:**
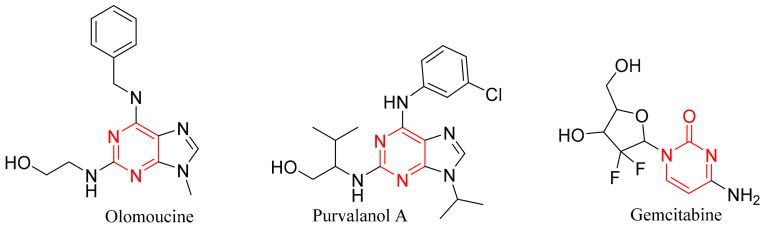
Pyrimidne-containing compounds that can act as anti-Bcl-2 and anti-apoptotic [28,43,45,46].

The pyrimidine derivatives now exhibit a number of biological activities, such as anti-inflammatory, anti-infectious, antidiabetic, as well as anticancer activity [41,42]. The most recognized drugs based on analogs of pyrimidines are anti-cancer, such as zidovudine, stavudine, 5-flurouracil, methotrexate; anti-infectious, such as sulfamethazine, trimethoprim; anti-psychotic, such as phenobarbital, primidone, risperidone, as well as other effective agents, such as imatinib, dasatinib, pazopanib, nilotinib, uramustine, tegafur, cytarabine [41,42]. Anti-breast cancer FAD-approved drugs bearing pyrimidine moiety are listed in Figure 4 [37,38,39,40]. The pyrimidine ring itself frequently augments the bioavailability of many drugs because of its capability to interact with various targets, such as bio-isosteres for phenyl and other aromatic systems through its formation of hydrogen bonds [42].

A lot of pyrimidine-containing compounds (as shown in Figure 4) were found to induce apoptosis, which inhibits the MCF7 proliferation by inducing caspase-3 and BAX, and by inhibiting anti-apoptotic proteins such as Bcl-2 and Mcl-1. So, the overexpression of p53 and BAX, and the inhibition of Bcl-2 gene enhances the death of breast carcinomas [28,43]. The major role of pyrimidines in cancer treatment originates from its resemblance to the nucleotides of the DNA and RNA [44], so it can interact via hydrophilic, aromatic and/or hydrogen bonds caused an increase in cytotoxicity%. Pyrimidines can also restrict the cell cycle by causing G0 phase apoptosis, and can cause cell cycle arrest in the G1/S phase. Some pyrimidine derivatives can induce the arrest of G2/M and S-phase in the MCF-7 cell cycle [43,45,46].

**Figure 5:**
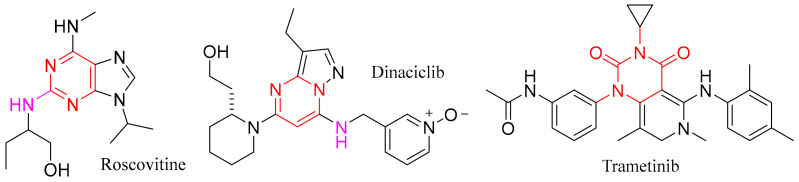
Bcl-2 and Mcl-1 inhibitor drugs on the market containing pyrimidine [25,47,48].

In the past few decades, a lot of Bcl-2 family inhibitors were introduced to the field such as Roscovitine, Venetoclax, Dinacilib, navitoclax and Trametinib [25,47,48], also there are a lot of other compounds still in clinical trials such as ABBV 467, A-1210477, AZD5991 and MIM1 [49,50,51], as shown in Figure 5.

Preparation of lead compounds **2**–**5**; the novel *S*-alkylated-4-amino-5-cyanopyrimidin-2-thiol (**2**) derivatives were prepared and were synthesized via the reaction of urea or thiourea with appropriate aromatic aldehydes and active methylene (malononitrile and/or ethyl cyanoacetate) in absolute ethanol at 120 °C to yield compounds **1a**–**d**, similar to what was previously prepared [52,53]. Thiopyrimidine derivatives were reacted with chlorinated derivatives in sodium carbonate as a basic medium, producing *S*-alkylated derivatives, as reported [54]. The prepared Compound **1b** was then reacted with appropriate chloro-compounds as 2-Nitrobenzyl chloride in dimethylformamide (DMF) containing catalytic amounts of sodium carbonate to obtain S-alkylated derivatives **2**. For the synthesis of new 4-amino-5-cyano pyrimidine derivatives **4a**–**c**, compounds **1a**–**c** were used as the starting materials as they were refluxed, separately, with phosphorus oxychloride to produce 4-chloro-derivatives **3a**–**c**. Based on the literature survey, 4-active-chloro-thiouracil derivatives **3a**–**c** were effectively reacted with laboratory-prepared heteroaromatic amine (different 2-amino-pyrrole-3-carbonitriles) which was previously reported by our research group [55,56]; this reaction yielded the corresponding 4-pyrrolo-5-cyanopyrimidines **4a**–**c**.

**Scheme 1:**
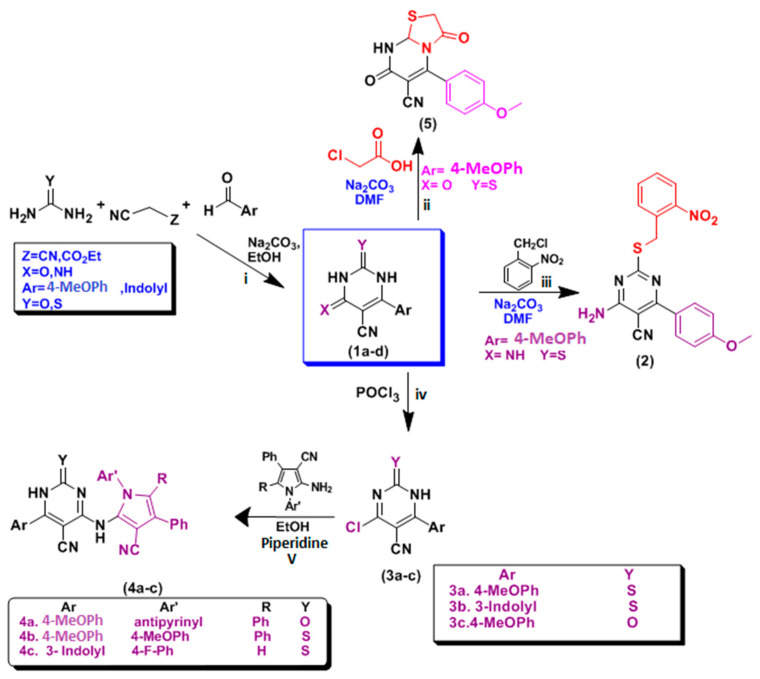
Synthesis of pyrimidine derivatives: (i) NaOH/EtOH, (ii) 2–Nitrobenzyl Chloride, Na_2_CO_3_, DMF (iii) α–chloroacetic acid, Na_2_CO_3_, DMF, (iv) POCl_3_, (v) aromatic amines, piperidine/EtOH.

Finally, Compound **5** was prepared by the reaction of compound **1d** with the appropriate chloro-compound as α-chloroacetic acid in the presence of DMF in basic media, expecting the synthesis of ordinary S-alkylated derivative but thiazolo[3,2-a]pyrimidine-6-carbonitrile **5** was obtained, as revealed in Scheme 1.

### 2.2. Biological Discussion

HBOC continues to be the most common cancer affecting women worldwide, maintaining its status as the most common tumor in females. For females, it is the second leading cause of deaths [57]. Differential expressions of the (Bcl-2) gene family has been distinguished in many cancer types. The primary indicators of apoptosis statute are members of the Bcl-2 protein family. By preserving the integrity of the mitochondrial membrane, Bcl-2 prevents apoptosis. It stops the release of several apoptogenic particles from mitochondrion as well as the oligomerization of BAX/BAK. Furthermore, by interacting with and deactivating BAX and other pro-apoptotic proteins, Bcl-2 may prevent apoptosis [58]. A pro-survival protein, (Mcl-1) mainly regulates the process of programmed cell death known as apoptosis. When Mcl-1 is overexpressed in breast cancer, it can prevent apoptosis, enabling cancer cells to avoid signals that would otherwise kill them and instead extending their lifespan. Additionally, Mcl-1 forms heterodimers with pro-apoptotic proteins like BIM, BAX and BAK to stop these factors from activating. The pro-apoptotic proteins are sequestered by this contact, which prevents them from initiating the mitochondrial apoptotic pathway [18].

#### 2.2.1. Cytotoxicity/Viability Assay for the Most Active Compounds

**Figure 6:**
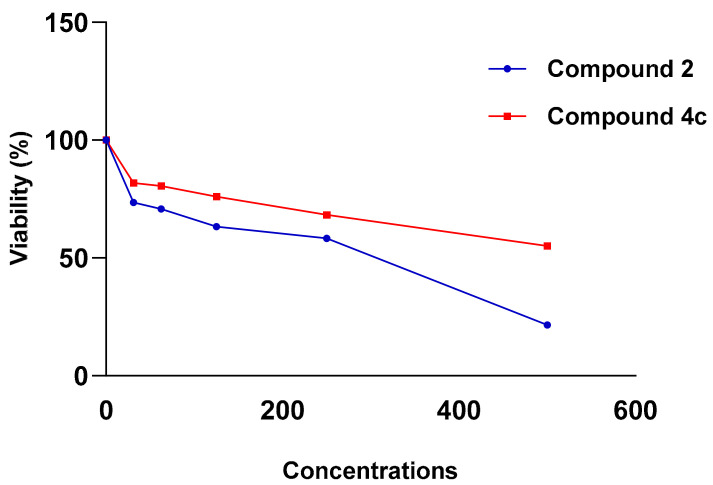
Viability (%) vs. Concentrations (µg/mL) chart of tested compounds **2** and **4c** in MCF-7 cell line.

**Figure 7:**
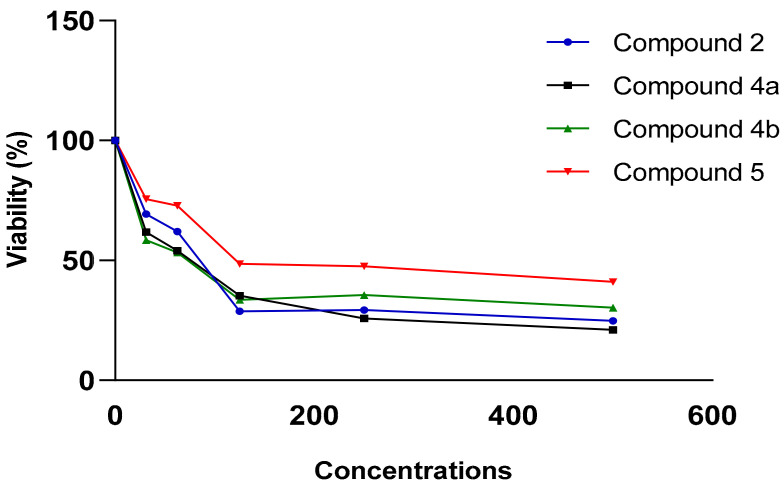
Viability (%) vs. concentrations (µg/mL) chart of tested compounds **2**, **4a**, **4b** and **5** in SKOV-3 cell line.

MTT assay was conducted to assess the anti-proliferative activity of the tested compounds against two different human cancer cell lines, MCF-7 (Human breast cancer cell line) and SCOV-3 (Ovarian cancer cell line). The concentrations of the compounds varied from 500 to 31.25 µg/mL. The IC_50_ was calculated based on inhibitor vs. response—variable slope (four parameters) using GraphPad Prism 9.0.0 (121) (Table 1) and corresponding graphs were produced (as shown in Figure 6 and Figure 7).

In the MCF-7 breast cancer cell line, compounds **2** and **4c** showed anti-proliferative activity with IC_50_ = 260.97 and 577.97 µg/mL, respectively. While compounds **2**, **4a**, **4b** and **5** showed superior anti-proliferative activity with IC_50_ = 78.79, 96.78, 76.19 and 90.83 µg/mL, respectively in SKOV-3 cell line.

#### 2.2.2. Cell Cycle Arrest and Apoptotic Cells Formation

##### Cell Cycle Arrest and Apoptosis Induction on MCF-7

**Figure 8:**
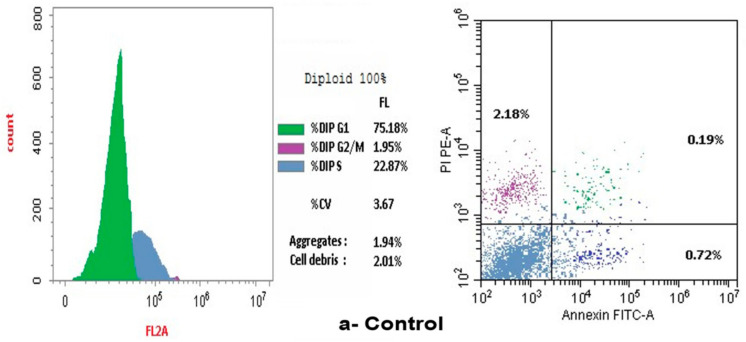

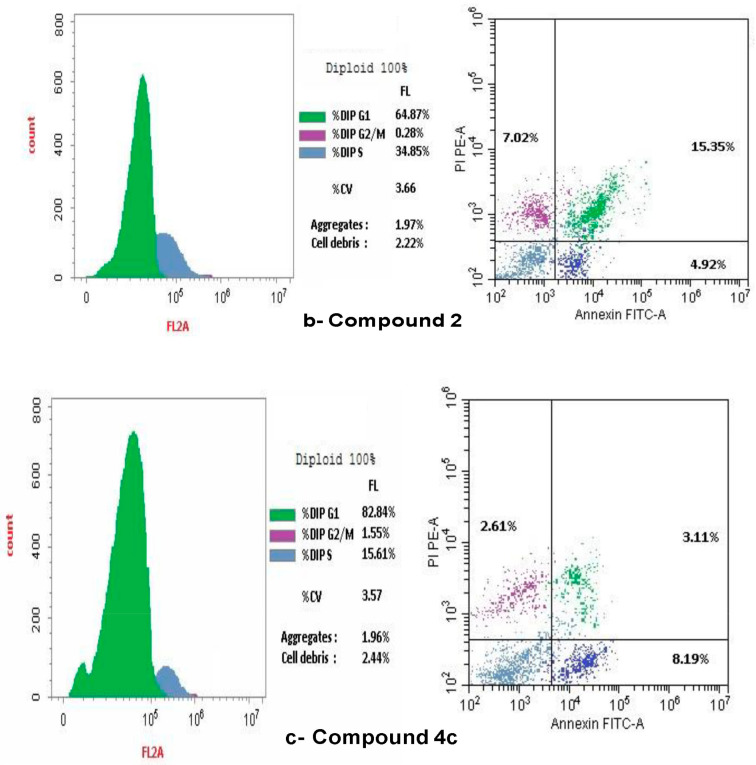
Effect of control, doxorubicin, compounds **2** and **4a** on cell cycle arrest in human SCOV-3 ovarian cancer cells measured by flow cytometry. (**a**) Control on cell cycle arrest in human SCOV-3 ovarian cancer cells. (**b**) Effect of compound **2** on the arrest of cell cycle in human SCOV-3. (**c**) Impact of **4c** compound on cell cycle arrest in human SCOV-3 ovarian cancer cells.

The present study employed flow cytometry to analyze the distribution of the cell cycle of MCF-7 cell line. Seventy-five point eighteen percent of MCF-7 cells are present in the G1 phase. In contrast, twenty-two point eighty-seven percent are in the S phase. The pre-G1 phase contains the majority of the cells, whereas a minority, particularly 1.95%, are present in the G2/M phases, as illustrated in Figure 8a.

The treatment of doxorubicin caused the arrest of the cell cycle at the G2/M stages, with 45.88% of cells in the G1 phase, however, a lesser percentage was in the G2/M phases (39.29%) and the S-phase (14.83%).

Additionally, the flow cytometric analysis of the cell cycle in cell lines of breast cancer treated with compound **2** revealed that 64.87% of cells were in the G1 phase, while a lesser percentage was present in the G2/M phases (0.28%) and the S phase (34.85%). Treatment with compound **2** induced cell cycle arrest during the S phase, as illustrated in Figure 8b.

However, the flow cytometric assessment of the cell cycle in breast cancer cell lines treated with compound **4c** revealed that 82.84% of the cells were mostly in the G1 phase. A lesser fraction of cells, 1.55%, was noted in the G2/M phases, whereas 15.61% were identified in the S phase. Treatment with compound **4c** induced cell cycle arrest specifically at the G1 phase, as illustrated in Figure 8c.

Cellular apoptosis was quantified after a 24 h period using flow cytometry. The research of BC cell lines demonstrated a heightened percentage of apoptotic cells (comprising late, early and necrotic cells) following Compounds **2** and **4c** treatment, in comparison to the control group. Figure 8 demonstrated that Compound **2** cells had a significantly higher percentage of apoptotic cells than Compound **4c** cells.

##### Cell Cycle Arrest and Apoptotic Cells Formation on SKOV-3

**Figure 9:**
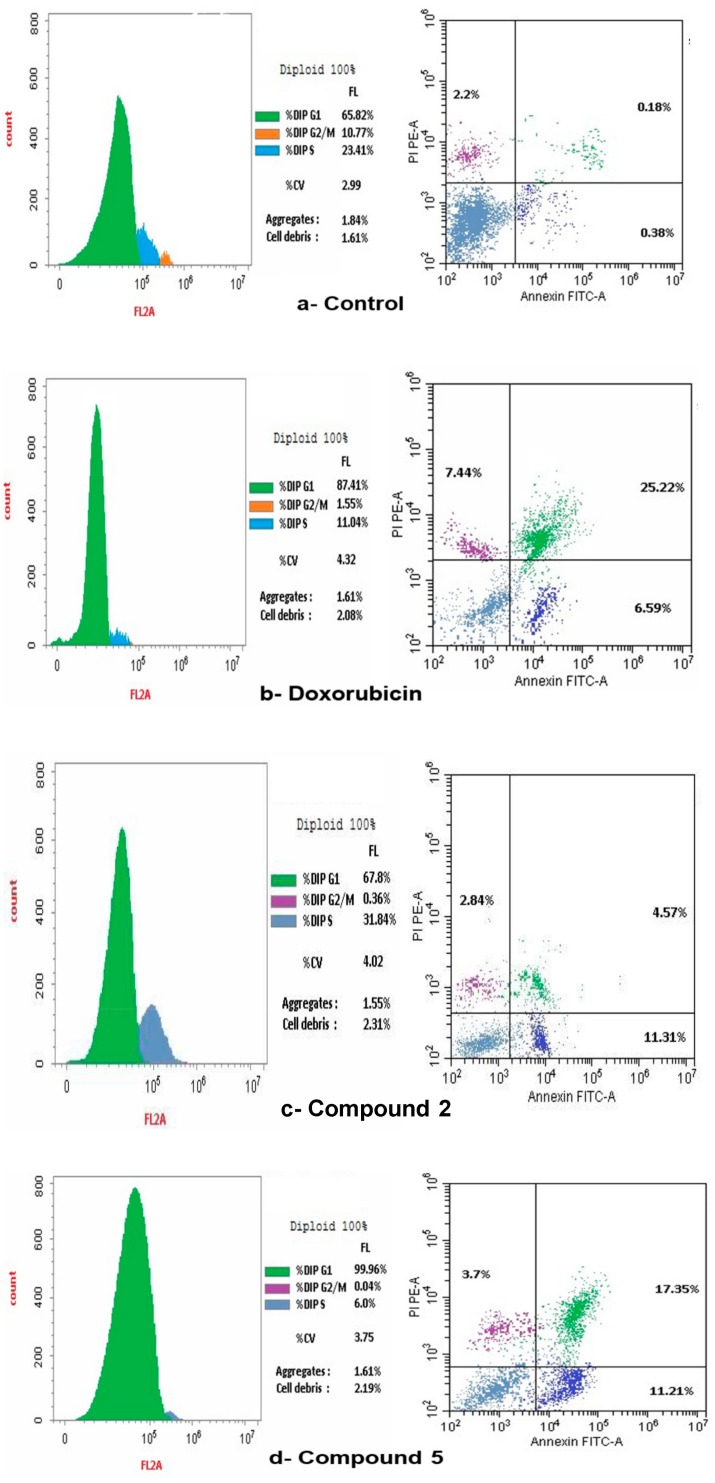

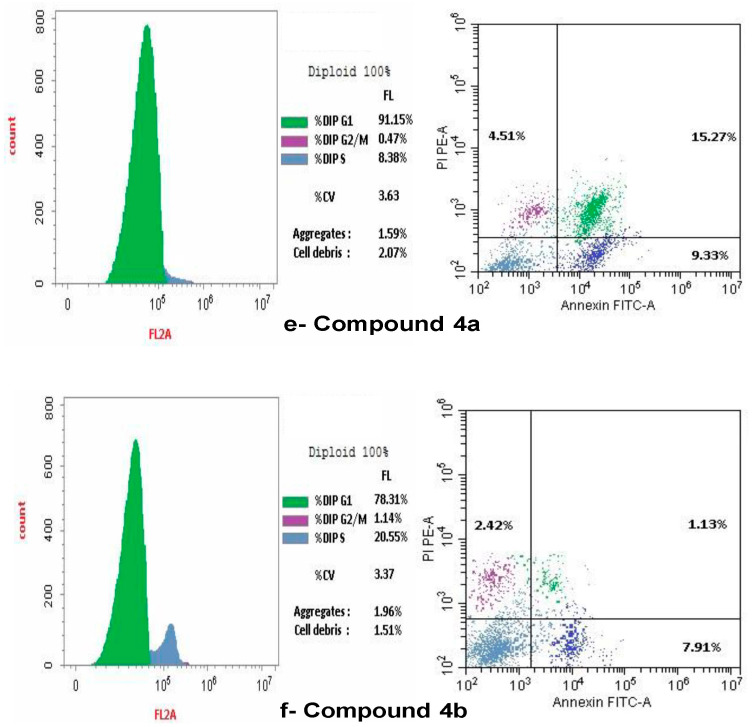
Effect of Control, doxorubicin, compounds **2**, **5**, **4a** and **4b** on cell cycle arrest in SCOV-3 measured by flow cytometry. (**a**) Control on cell cycle arrest in SCOV-3 cells. (**b**) Effect of doxorubicin on cell cycle arrest in human SCOV-3 cells. (**c**) Effect of compound **2** on the arrest of cell cycle in SCOV-3. (**d**) Impact of compound **5** on cell cycle arrest in human SCOV-3 cells. (**e**) Effect of compound **4a** on cell cycle arrest in human SCOV-3. (**f**) Impact of compound **4b** on cell cycle arrest in human SCOV-3 cells.

The present study employed flow cytometry to analyze the cell cycle distribution in SKOV-3. The G1 phase shows: 65.82% of SKOV-3, and 23.41% at the S phase. The predominant proportion of cells resides in the pre-G1 phase, with 10.77% occupying the G2/M phases (Figure 9a). A flow cytometric study of the cell cycle in SKOV-3 treated with doxorubicin indicated: 87.41% in the G1 phase, but 1.55% in the G2/M phase and 11.04% in the S phase, therefore the arrest of the cell cycle was defined at the G1 phase, as illustrated in Figure 9b. A flow cytometric cell cycle examination of compound **2** on ovarian cancer cell lines indicated that 67.8% of cells were in the G1 phase, however, a smaller percentage was in the G2/M (0.36%) and S phases (31.84%). Figure 9c illustrated that compound **2** resulted in the cell cycle being halted at the S phase. Additionally, the flow cytometric study of the cell cycle in ovarian cancer cell lines treated with compound **5** demonstrated that 99.96% of cells were in the G1 phase, while a smaller percentage were in the G2/M (0.04%) and S phases (6.0%). Figure 9d illustrated that compound **5** resulted in the cell cycle being halted at the G1 phase. A flow cytometric cell cycle examination of compound **4a** on SCOV-3 cell lines indicated that 91.15% of cells were in the G1 phase, while a smaller percentage was in the G2/M (0.47%) and S phases (8.38%). Figure 9e illustrates that the administration of chemical 4a resulted in the cell cycle being halted at the G2/M phases. A flow cytometric analysis of compound **4b** on ovarian cancer cell lines indicated that 78.31% of cells were in the G1 phase, while a smaller percentage were in the G2/M phases (1.14%) and the S phase (20.55%). Figure 9f illustrated compound **4b** resulted in the arrest of cell cycle at the S phase.

Flow cytometry was occupied to assess cellular apoptosis following a 24 h period. Ovarian cancer cell lines were treated with compounds **2**, **4a**, **4b** and **5**, with the conventional medication doxorubicin. Apoptotic cell percentage, encompassing early, late, and necrotic cells, rose in comparison to the control Figure 9a–f.

#### 2.2.3. RT PCR

**Figure 10:**
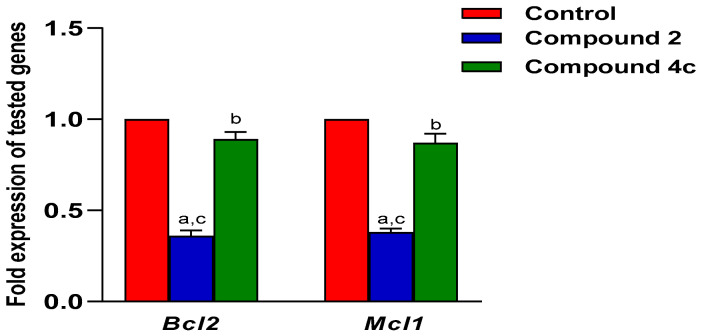
The Bcl-2 and Mcl-1 gene expression measured by RT-qPCR in MCF-7 cell line. Mean ± SEM is the statistical distribution used to represent values. A *p*-value <0.05 was interpreted as demonstrating statistical significance. a: significant when compared to control at *p* < 0.05. b: significant when compared to Compound **2** at *p* < 0.05. c: significant when compared to Compound **4c** at *p* < 0.05.

It is crucial to investigate the potential molecular mechanisms following the treatment of MCF-7 and SKOV-3 cell lines with the tested compounds. Consequently, it was particularly pertinent to examine the patterns of the Mcl-1 and Bcl-2 genes’ expression. Table 2 presents the gene expression levels for Mcl-1 and Bcl-2 in both cell lines. In the case of MCF-7, the results indicated a significant decrease in Bcl-2 and Mcl-1 genes expression in the group administered compound **2**, relative to the control and the group treated with compound **4c**. Furthermore, there was a reduction in gene expression in the group administered with compound 4c relative to the control. However, compound **4c** exhibited a notable rise when compared to compound **2** as illustrated in Figure 10.

**Figure 11:**
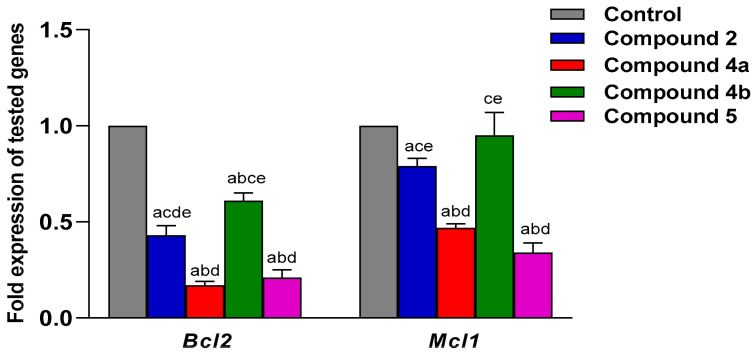
The Bcl-2 and Mcl-1 gene expression measured by RT-qPCR in SKOV-3 cell line. Mean ± SEM is the statistical distribution used to represent values. A *p*-value < 0.05 was interpreted as demonstrating statistical significance. a: significant when compared to control at *p* < 0.05. b: significant when compared to Compound **2** at *p* < 0.05. c: significant when compared to Compound **4a** at *p* < 0.05. d: significant when compared to Compound **4b** at *p* < 0.05. e: significant when compared to Compound **5** at *p* < 0.05.

The same scenario occurred in the SKOV-3, where all tested compounds successfully induced downregulation of the two genes. However, compounds **4a** and compound **5** exhibited the greatest reduction in expression for both genes, as demonstrated in Figure 11. The fold changes in the expression of the selected genes were determined via univariate analysis one-way ANOVA followed by multiple comparison test. The data are stated by the mean ± SEM. All graphs were plotted using GraphPad Prism 9.0.0 (121).

### 2.3. Molecular Docking Study

According to the results of the biological analysis, a molecular docking research was conducted to investigate the further potential mode of action of the most promising evaluated compounds (**2** and **4c**).

**Figure 12:**
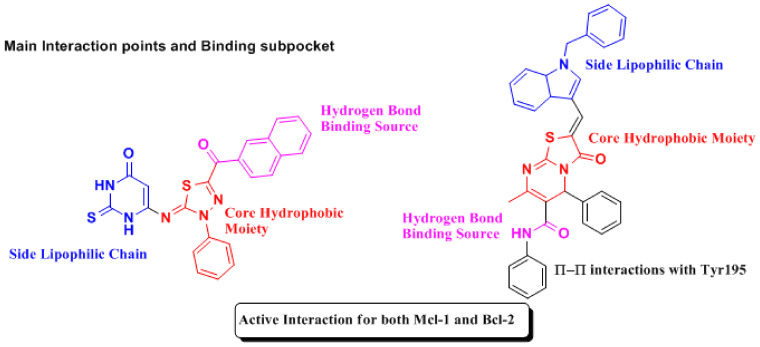
Structural Features of Mcl-1 and Bcl-2 inhibitors [47,63].

**Figure 13:**
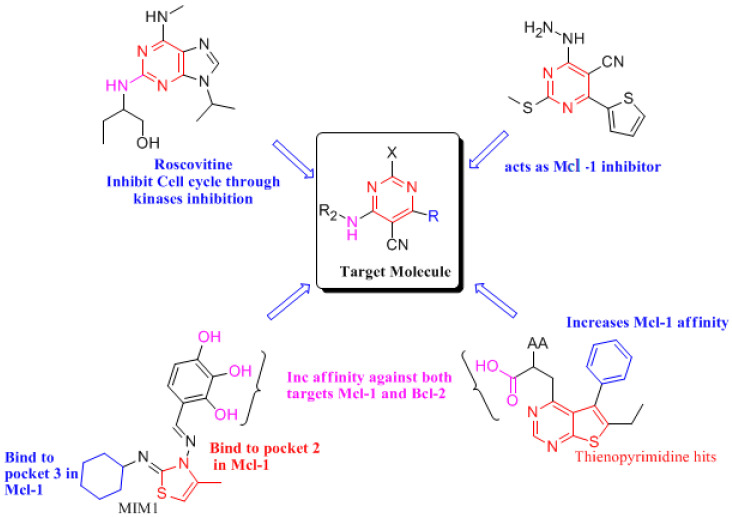
Relation between target molecules and Bcl-2, Mcl-1 inhibitors [50,64].

To understand the main drugs mode of action with the Bcl-2 and Mcl-1, the interaction with main active site must be well known. The most effective drug and Bcl-2/Mcl-1 interaction depends on the morphology of the receptors, and where they bind, it was found that the Bcl-2 hydrophobic pockets [59,60] (protein hot spots) were found to be P2 and P4 pockets [61]. However, with Mcl-1 [59], the most common interaction was the interaction with acidic hydrophilic moiety Arg263, aside from hydrophobic-hot spot, P2 and P3 pockets [62]. The main indication is that, if a compound can fit into P2, P3 and P4, it has a strong potential to have a dual action on Bcl-2/Mcl-1 inhibitions. Based on the morphology of the receptors, we were willing to synthesize pyrimidine derivatives (as shown in Scheme 1) with promising anti-breast cancer activity, which has a dual effect against Bcl-2/Mcl-1 and can also cause cell cycle arrest. This was carried out through the synthesizing of some compounds with similar structural features (Figure 12 and Figure 13) to the known anti- Bcl-2/Mcl-1 drugs and try to enhance its binding and selectivity to either Mcl-1 or Bcl-2 and its interference with the cell cycle, for example by exchanging the cyclohexane in MIM-1 with a large lipophilic molecule, as in compound **4c**, giving a better chance for fitting in the pockets and increasing the Mcl-1 affinity or by increasing hydrogen bonds’ interaction by introducing a nitro group, as in compound **2**.

**Figure 14:**
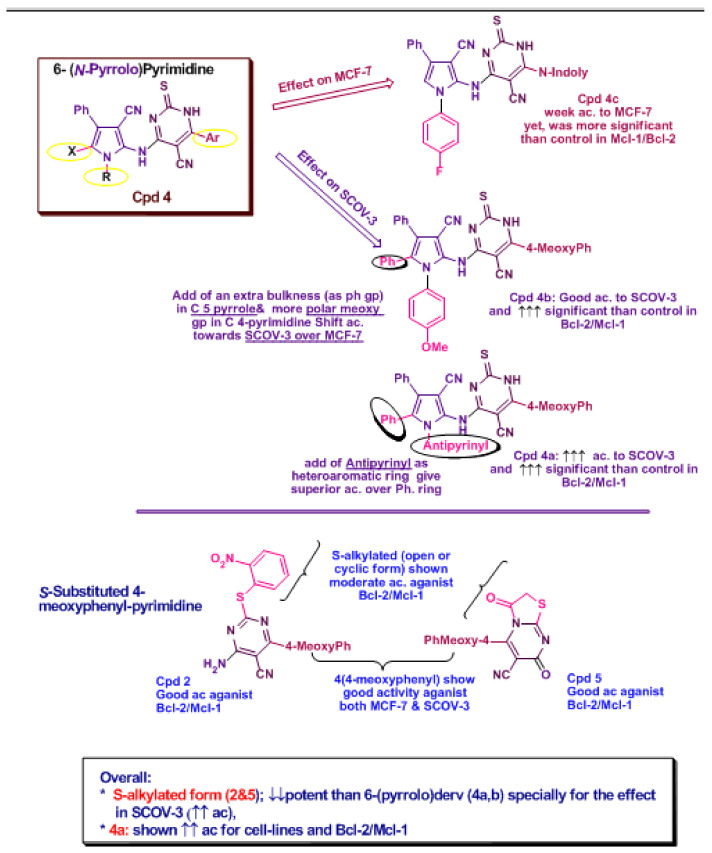
Structure Co-relation for the most active compounds (**2**, **4a**, **4b** and **5**) in both tested cell lines.

The docking studies were carried out based on the findings and labor of the researcher’s former works [65,66,67,68] and our previous work [66,69,70]. The docking analysis verified the biological data indicated above, and the accompanying Figure 14 showed the structure correlation of the majority of active chemicals.

**Figure 15:**
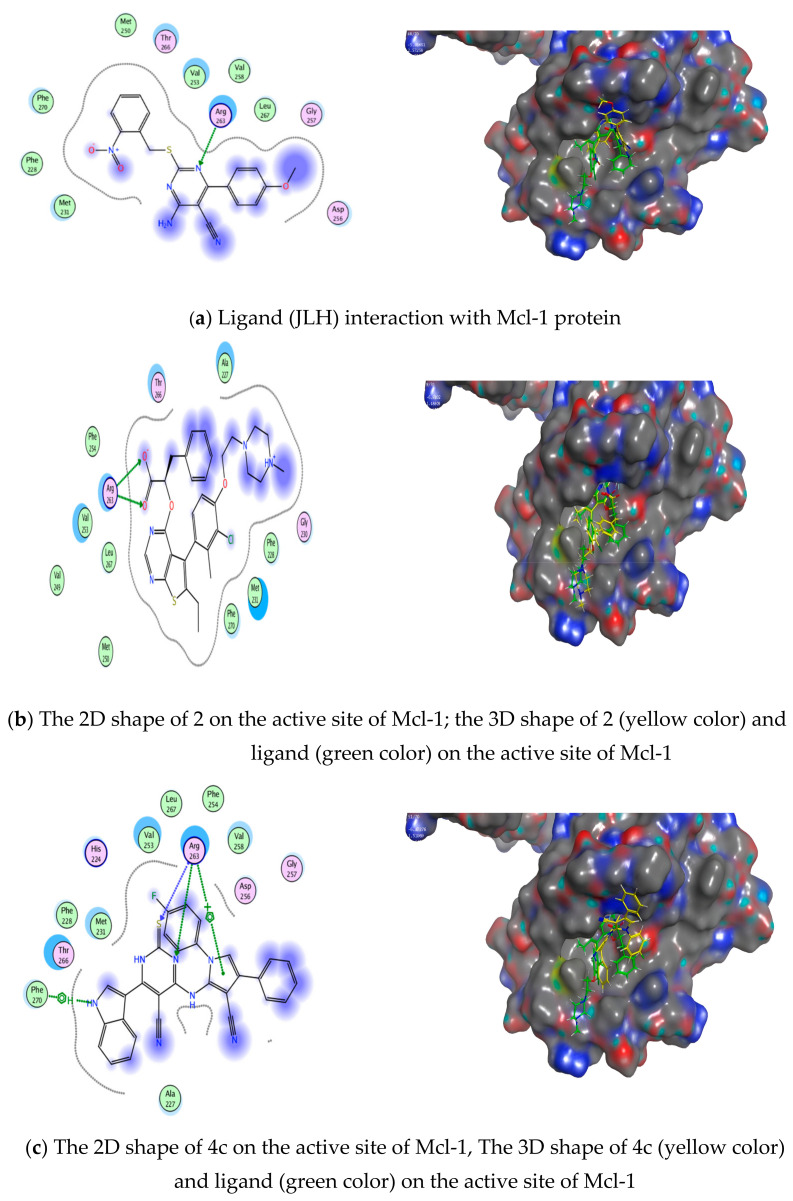
Ligand compounds **2** and **4c** interactions with Mcl−1 protein: (**a**) ligand (6QYO) interaction with Bcl-1 protein, (**b**) drug **2** interaction with Bcl−1 protein, (**c**) drug **4c** interaction with Bcl-1 protein.

We can summarize the outcomes of the docking study on Mcl-1 protein active sites: the results showed that both compounds **2** and **4c** interacted with the hotspots of the protein through lying in pockets 2 and 3, and the nitrogen atom of the pyrimidine in both **2** and **4c** performed H-bonds with Arg263, which is crucial for binding to the protein. The compounds were compared to the reference ligand JLH [59] and were compatible with the ligand results (as shown in Table 3 and Figure 15).

**Figure 16:**
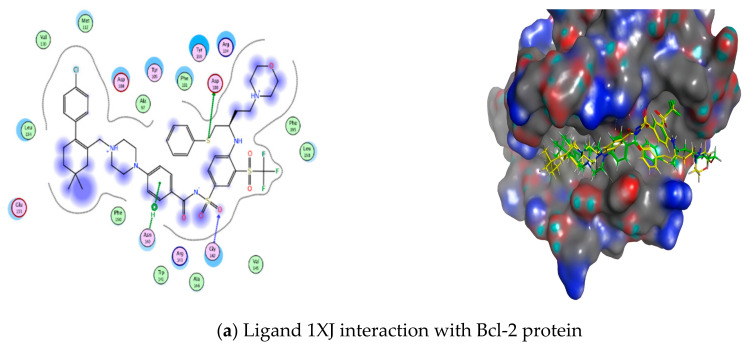

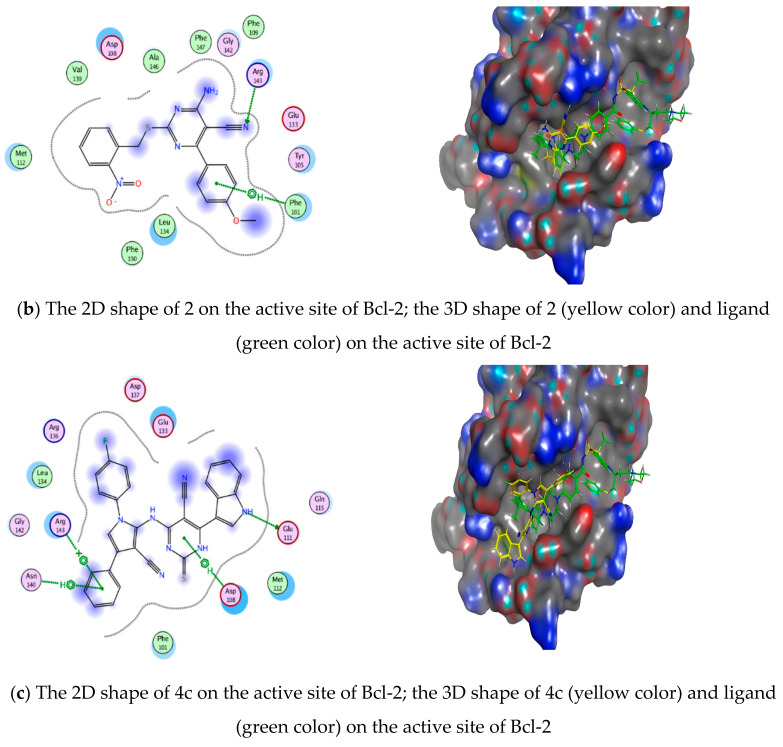
Ligand, compound **2** and **4c** interactions with Bcl-2 protein: (**a**) Ligand 1XJ interaction with Bcl-2 protein, (**b**) Drug **2** interaction with Bcl-2 protein, (**c**) Drug **4c** interaction with Bcl-2 protein.

However, the results of the docking study on Bcl-2 protein revealed that both compound **2** and **4c** formed hydrogen bonds with Arg143. Also, both of them lied in the hotspot of Bcl-2 protein, and they both lied in pockets 2 and 3, but compound **2** did not only fit in pockets 2 and 3—it also fitted in pocket 4, proving its higher probability in having dual action against both Bcl-2 and Mcl-1 proteins and its higher efficacy against MCF-7 cell line. Their docking scores were calculated and compared to the reference ligand Navitoclax 1XJ [59,60] and they showed well-matched interactions in Table 4 and Figure 16.

## 3. Materials and Methods

### 3.1. Synthesis of Lead Compounds

All melting points were uncorrected and determined using Electro-thermal IA 9100 apparatus (Shimadzu, Kyoto, Japan). IR spectra were recorded as potassium bromide pellets on a Perkin-Elmer 1650 spectrophotometer (Waltham, MA, USA) and the resulted values were expressed in cm^−1^. IR analysis was carried out at Faculty of Science, Helwan University. ^1^H-NMR spectra were recorded in DMSO-d_6_ on a Varian Mercury (400 MHz) spectrometer and chemical shifts were expressed as ppm, using TMS as an internal reference. ^1^H-NMR analysis was carried out in “Center for Drug Discovery Research and Development” at Faculty of Pharmacy, Ain-Shams University, Cairo, Egypt. Mass spectra were carried out on 70 eV EI MS-1000 EX, at “The Regional Center for Mycology and Biotechnology”, Al-Azhar University, Cairo, Egypt. The elemental analysis of the new compounds was carried out in “Micro analytical Center” at the faculty of science, Cairo University, Giza, Egypt. Chemical reactions were monitored using Thin Layer Chromatography (TLC). TLC was performed on pre-coated silica gel (Merck, Darmstadt, Germany), in the appropriate solvent system and UV-light was used for spots visualization. All the prepared compounds are new except **1a**–**d** and **3a**, **b** [53,71,72,73].

General procedure for the synthesis of 6-(substituted)-pyrimidine-5-carbonitriles **1a**–**d**:

Compounds **1a**–**d** were prepared as reported [52] by the reaction of equimolar amounts of urea or thiourea, appropriate aromatic aldehydes, and active methylene (namely, malononitrile and ethylcyanoacetate) in absolute ethanol (35 mL). The reaction mixture was heated under reflux. The reaction was monitored by TLC. Reaction mixture was cooled, poured onto ice/water then acidified with few drops of HCl. The formed precipitate was collected, washed with water, then recrystallized from ethanol to give compounds **1a**–**d**

*6-(4-methoxyphenyl)-4-oxo-2-thioxo-1,2,3,4-tetrahydropyrimidine-5-carbonitrile [71]* (**1a**): Yield: 40%, m.p: 237–239 °C As reported [71]

*6-(1H-indol-3-yl)-4-oxo-2-thioxo-1,2,3,4-tetrahydropyrimidine-5-carbonitrile* (**1b**): [53] Yield 60%; m.p.: 195–198 °C As reported [71]

*6-(4-methoxyphenyl)-2,4-dioxo-1,2,3,4-tetrahydropyrimidine-5-carbonitrile* (**1c**): [72] Yield (42%), m.p: 227–229 °C As reported [72]

*4-amino-6-(4-methoxyphenyl)-2-thioxo-1,2-dihydropyrimidine-5-carbonitrile* (**1d**):

Yield: 50%, m.p: 260–262 °C As reported [73].

Preparation of 4-amino-6-(4-methoxyphenyl)-2-((2-nitrobenzyl)thio)pyrimidine-5-carbonitrile (**2**):

A mixture of 4-aminopyrimidine **1b** (0.01 mole) and 2-nitrobenzyl chloride (0.01 mole) in 30 mL DMF with catalytic amount of Na_2_CO_3_ (1.06 g, 0.01 Mole) was stirred for 6 h at room temperature. Then the solution was diluted with cold water; 30 mL 95% ethanol was added. The reaction mixture was then heated with stirring to evaporate any excess DMF, the solid is filtered off and recrystallized from ethanol to give product **2**.

Yield: 40%; m.p.: 165–167 °C; IR (KBr) ν (cm^−1^): 3415, 3392 (NH_2_), 2249 (C≡N), 1352, 1541 (NO_2_), 1540 (C=N); MS (EI) *m*/*z*: 393.27[M]^+.^ (41.54%); H^1^-NMR (DMSO-d_6_) δ (ppm): 3.87 (s, 3H, OCH_3_), 4.76 (s, 2H, SCH_2_), 7.13–8.26 (m, 10H: 8H Ar-H + 2H NH_2_ D_2_O exchangeable); Anal. Calcd. for C_19_H_15_N_5_O_3_S (393.42): C, 58.01; H, 3.82; N, 17.81; S, 8.14%. Found: C, 58.14; H, 4.05; N, 17.73; S, 8.15%.

General procedure for preparation of 4-chloro-6-substitutedpyrimidine-5-carbonitriles (**3a**–**c**) [72]

4-aminopyrimidines **1a**–**c** (0.01 mole) were heated, independently, under reflux in phosphorus oxychloride (30 mL) for 12 h. The solution was cooled then poured with stirring onto ice/water. The formed precipitate was filtered off, dried, and recrystallized from ethanol to give the target compounds **3a**–**c**

*4-chloro-6-(4-methoxyphenyl)-2-thioxo-1,2-dihydropyrimidine-5-carbonitrile [72]* (**3a**): Yield: 54%; m.p.: 172–174 °C; as reported [72]

*4-chloro-6-(1H-indol-3-yl)-2-thioxo-1,2-dihydropyrimidine-5-carbonitrile [53]* (**3b**)

Yield: 50%; m.p.: 150–152 °C; as reported [53]

*4-chloro-6-(4-methoxyphenyl)-2-oxo-1,2-dihydropyrimidine-5-carbonitrile* (**3c**):

Yield: 52%; m.p.: 156–158 °C; IR (KBr) cm^−1^: 3140 (NH), 2200 (C≡N), 1740 (C=O), 1624 (C=N); MS (EI) *m*/*z*: 261 [M]^+.^ (31%), 263 [M+2] (10.11%); H^1^-NMR (DMSO-d_6_) δ (ppm): 3.62 (s, 3H, OCH_3_), 7.63–8.18 (m, 5H: 4H Ar-H + 1H NH D_2_O exchangeable); Anal. Calcd. for C_12_H_8_ClN_3_O_2_ (261.66): C, 55.08; H, 3.08; N, 16.06%. Found: C, 55.19; H, 2.95; N, 16.10%.

General procedure for preparation of 4-(substituted) amino-dihydropyrimidine-5-carbonitrile (**4a**–**c**)

A mixture of equimolar amounts of 4-chloropyrimidines **3a**–**c** and appropriate 2-amino-pyrrole-3-carbonitriles in 30 mL 95% ethanol was heated under reflux in the presence of catalytic amount of piperidine for 48 hrs. The product is separated by pouring the mixture over ice/water and treatment with conc HCL (3 mL), then filtered off and washed by water, the ppt is then recrystallized from ethanol to yield compounds **4a**–**c**.

*4-((3-cyano-1-(1,5-dimethyl-3-oxo-2-phenyl-2,3-dihydro-1H-pyrazol-4-yl)-4,5-diphenyl-1H-pyrrol-2-yl)amino)-6-(4-methoxyphenyl)-2-oxo-1,2-dihydropyrimidine-5-carbonitrile* (**4a**): Yield: 40%; m.p.: 198–200 °C; IR (KBr) ν (cm^−1^): 3433, 3315 (NH), 2285, 2215 (C≡N), 1706, 1684 (C=O); MS (EI) *m*/*z*: 670.73[M]^+.^ (28%); ^1^H NMR (DMSO-d_6_): δ (ppm): 2.83 (s, 3H, CH_3_), 3.14 (s, 3H, CH_3_), 3.78 (s, 3H, OCH_3_), 7.06–8.11 (m. 21H: 19H, Ar-H + 1H NH, D_2_O exchangeable + 1H NH, D_2_O exchangeable); Anal. Calcd. for C_40_H_30_N_8_O_3_ (670.73): C, 71.63; H, 4.51; N, 16.71%. Found: C, 71.54; H, 4.73; N, 16.55%.

*4-((3-cyano-1-(4-methoxyphenyl)-4,5-diphenyl-1H-pyrrol-2-yl)amino)-6-(4-methoxyphenyl)-2-thioxo-1,2-dihydropyrimidine-5-carbonitrile* (**4b**):

Yield: 61%; m.p.: 182–184 °C; IR (KBr) ν (cm^−1^): 3298, 3240 (NH), 2273, 2223 (C≡N); MS (EI) *m*/*z*: 606[M]^+.^ (38.9%); ^1^H-NMR (DMSO-d_6_, 400 MHz) δ (ppm): 3.34 (s, 3H, OCH_3_), 3.82 (s, 3H, OCH_3_), 6.91–8.40 (m. 20H: 18H, Ar-H + 1H NH, D_2_O exchangeable + 1H NH, D_2_O exchangeable); Anal. Calcd. for C_36_H_26_N_6_O_2_S (606.704): C, 71.27; H, 4.32; N, 13.85%. Found: C, 71.50; H, 4.39; N, 13.77%.

*4-((3-cyano-1-(4-fluorophenyl)-4-phenyl-1H-pyrrol-2-yl)amino)-6-(1H-indol-3-yl)-2-thioxo-1,2-dihydropyrimidine-5-carbonitrile* (**4c**):

Yield: 42%; m.p.: 145–147 °C; IR (KBr) ν (cm^−1^): 3329–3265 (NH), 3052, 2922 (CH), 2278, 2225 (C≡N); MS (EI) *m*/*z*: 527[M]^+.^ (85.85%); H NMR (DMSO-d_6_, 400 MHz) δ (ppm): 7.03–8.20 (m, 16H H: 15H, Ar-H + 1H NH, D_2_O exchangeable), 8.92 (1H, NH, D_2_O exchangeable), 9.91 (1H, NH, D_2_O exchangeable); Anal. Calcd. for C_30_H_18_FN_7_S (527.58): C, 68.30; H, 3.44; N, 18.58%. Found: C, 68.52; H, 3.37; N, 18.70%.

Preparation of 5-(4-methoxyphenyl)-3,7-dioxo-2,3-dihydro-7H-thiazolo[3,2-a]pyrimidine-6carbonitrile (**5**)

A mixture of 4-aminopyrimidine **1b** (0.01 mole) and α-chloroacetic acid (0.01 mole) in 30 mL DMF with catalytic amount of Na_2_CO_3_ (1.06 g, 0.01 Mole) was stirred for 6 h at room temperature. The solution was diluted with cold water; 30 mL 95% ethanol was added. The reaction mixture was then heated with stirring to evaporate any excess DMF, the solid is filtered off and recrystallized from ethanol to give product **5**.

Yield: 46%; m.p.: 303–305 °C; IR (KBr) ν (cm^−1^): 3254 (N-H), 2352 (C≡N), 1693, 1682 (C=O); MS (EI) *m*/*z*: 300 [M]^+^ (55.78%); H^1^-NMR (DMSO-d_6_) δ (ppm): 3.75 (s, 3H, OCH_3_), 3.82 (s, 2H, SCH_2_), 4.76 (s, 1H, CH), 7.0–7.87 (m, 5H H: 4H, Ar-H + 1H NH, D_2_O exchangeable); Anal. Calcd. for C_14_H_11_N_3_O_3_S (301.32): C, 55.81; H, 3.68; N, 13.95; S, 10.64%. Found: C, 55.66; H, 3.56; N, 13.72; S, 10.54%.

### 3.2. Biological Assay

The additional data included the experimental materials and procedures for every biological test, such as cell culture, cytotoxicity assay, cell cycle and apoptosis detection via flow cytometry, and analysis of Bcl-2 and Mcl-1 gene expression with RT-qPCR (Table 5), are provided in the Supplementary Data (as S1) [74,75].

### 3.3. Docking Studies

The molecular operating environment (MOE) software was used for all docking experiments in this work, and the additional data contained information on every step of the docking procedure are provided in the Supplementary Data (as S2).

The molecular operating environment (MOE) software was used to dock our most active derivatives (**2** and **4c**) into the 3D forms of both Bcl-2 and Mcl-1 with the proper ligands: the crystal structures of Bcl-2 (PDB:4LVT) at 2.6 Å resolution, and the crystal structure of Mcl-1 (PDB code: 6QYO)) at 2.6 Å resolution. As a representation of the conformational configurations with the most favorable binding energy, the predicted binding of the target derivatives to each Bcl-2 and Mcl-1 active pocket was found to be the best categorized scoring function. (ΔE).

## 4. Conclusions

**Figure 17:**
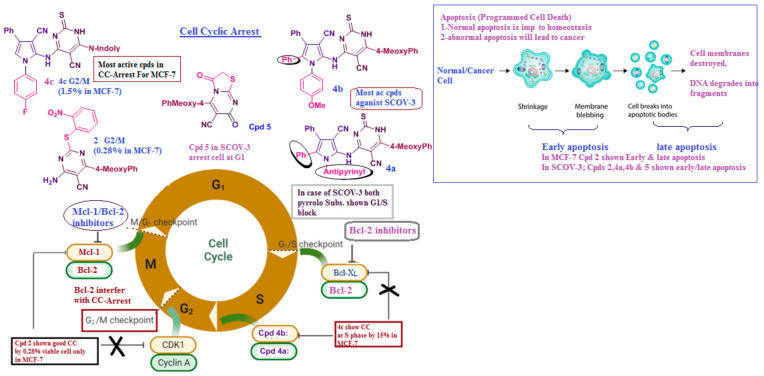
Summary for all bioactive compounds’ main effects on apoptosis and cell cyclic arrest.

Despite having modest cytotoxic activity (IC50), compounds **2** (in SCOV-3/MCF-7) and **4b**, **5** (in SCOV-3) were able to block the cell cycle in the S and G2/M phases, produce the highest percentage of apoptosis, and have the strongest inhibitory effect on the expression of the Mcl-1/Bcl-2 gene. As a result of cell cycle arrest and triggered apoptosis, **4b**, the pyrrole substituted pyrimidine, **2** as s-alkylated, and **5** as thiazole pyrimidine have shown early indications of potential anticancer action. They may also serve as potential anti-Mcl-1/Bcl-2 medicines. Confirming the review of the literature on primary bioactive pyrimidines as a model scaffold for the production of new anti-cancer medications, As revealed in Figure 17, MCL-1 is an anti-apoptotic member of the Bcl-2 family, while the Bcl-2 gene is known for its role in regulating apoptosis, and its overexpression can contribute to cancer cell survival. When Bcl-2 and MCL-1 are downregulated in treated groups, it typically indicates an increase in apoptosis and respond better to the targeted treatments.

## Figures and Tables

**Table 1:** The half-maximal inhibitory (IC_50_) concentration of tested compounds.

Compounds	IC_50_ (μg/mL) (SKOV-3)	IC_50_ (μg/mL) (MCF-7)
Compound **2**	78.79	260.97
Compound **4a**	96.78	-
Compound **4b**	76.19	-
Compound **4c**	-	577.97
Compound **5**	90.83	-
Doxorubicin	19.37 ± 0.7	38.75 ± 1.4

**Table 2:** The Bcl-2 and Mcl-1 gene expression mRNA fold change measured by RT-qPCR in various studied groups.

	MCF-7 Cell Line		SKOV-3 Cell Line	
Groups	Bcl-2	Mcl-1	Bcl-2	Mcl-1
Control	1 ± 0.00	1 ± 0.00	1 ± 0.00	1 ± 0.00
Compound **2**	0.36 ± 0.03 ^a, c^	0.38 ± 0.02 ^a, c^	0.43 ± 0.05 ^a, c, d, e^	0.79 ± 0.04 ^a, c, e^
Compound **4a**	-	-	0.17 ± 0.02 ^a, b, d^	0.47 ± 0.02 ^a, b, d^
Compound **4b**	-	-	0.61 ± 0.04 ^a, b, c, e^	0.95 ± 0.12 ^c, e^
Compound **4c**	0.89 ± 0.04 ^b^	0.87 ± 0.05 ^b^	-	-
Compound **5**	-	-	0.21 ± 0.04^a, b, d^	0.34 ± 0.05 ^a, b, d^

a: significant when compared to control at *p* < 0.05. b: significant when compared to Compound **2** at *p* < 0.05. c: significant when compared to Compound **4c** at *p* < 0.05. d: significant when compared to Compound **4b** at *p* < 0.05. e: significant when compared to Compound **5** at *p* < 0.05.

**Table 3:** Docking results of compounds **2**, **4c** and ligand JLH with Mcl-1.

Compounds	Docking Score (S)	RMSD	E-Score 1	E-Score 2	Binding Interactions
Ligand JLH	−6.9102	1.4881	−9.9729	−6.9102	(O-Arg263)(H-b)
Compound **2**	−5.56651	2.57258	−8.31193	−5.56651	(N-Arg263)(H-b)
Compound **4c**	−6.37276	1.51069	−8.44352	−6.37276	(S-Arg263)(H-b) (N-Arg263)(H-b) (pyrrole-Arg263)(H-Pi-) (N-Phe270)(H-b)

**Table 4:** Docking results of compounds **2**, **4c** and ligand 1XJ with Mcl-1.

Compound	Docking Score (S)	RMSD	E-Score 1	E-Score 2	Binding Interactions
Ligand 1XJ	−8.7886	2.0588	−8.8739	−8.7886	(S-Asp100) (H-b) (O-Gly142) (H-b) (Benzene-Asn140) (H-Pi)
Compound **2**	−5.7703	2.3114	−9.2490	−5.7703	(N-Arg1430 (H-b) (Benzene-Phe101) (H-Pi)
Compound **4c**	−6.2246	1.6794	−8.9713	−6.2246	(Benzene-Arg143)(Pi-cation) (Benzene-Asn140) (H-Pi) (Benzene-Asp108) (H-Pi)

**Table 5:** Bcl-2, Mcl-1 and GAPDH gene primer sequences.

Target	Sequence
Bcl-2	F 5′-ATCGCCCTGTGGATGACTGAGT-3′ R 5′-GCCAGGAGAAATCAAACAGAGGC-3′
Mcl-1	F 5′-CCAAGAAAGCTGCATCGAACCAT-3′ R 5′-CAGCACATTCCTGATGCCACCT-3′
GAPDH	F 5′-GTCTCCTCTGACTTCAACAGCG-3′ R 5′-ACCACCCTGTTGCTGTAGCCAA-3′

## Data Availability

All the data are original, and are present in the article and supplementary data.

## Supplementary Materials

The following supporting information can be downloaded at: https://www.mdpi.com/article/10.3390/molecules30071453/s1. S1: Biological Assay; Table S1: SKOV cell lines & Mcf-7 Cell lines; S2: Docking Studies; S3: Specroscopic analysis; Figure S1a: C13 of Compound **2**; Figure S1b: C13 of Compound (**4b**); Figure S1c: Mass spectrum of compound (**2**); Figure S1d: ^1^H-NMR spectrum of compound **2** DMSO-d_6_; Figure S1e: 1H-NMR spectrum of compound **2** D_2_O; Figure S1f: Mass spectrum of compound (**4a**); Figure S1g: 1H-NMR spectrum of compound **4a** DMSO-d_6_; Figure S1h: 1H-NMR spectrum of compound **4a** D_2_O; Figure S1i: Mass Spectrum of compound **4b**; Figure S1j: ^1^H-NMR spectrum of compound **4b**; Figure S1k: 1H-NMR spectrum of compound **4b** D_2_O; Figure S1l: Mass Spectrum of Compound **4c**; Figure S1m: 1H-NMR spectrum of compound **4c** DMSO-*d*_6_; Figure S1n: 1H-NMR spectrum of compound **4c** D_2_O; Figure S1o: Mass spectrum of compound **5**; Figure S1p: ^1^H-NMR spectrum of compound **5** DMSO-d_6_.
