# Supplementary material for: Novel Cyanopyrimidine Derivatives as Potential Anticancer Agents
Source: Molecules. 2025 Mar 25;30(7):1453. doi: 10.3390/molecules30071453 (PMC11990787; doi:10.3390/molecules30071453)
Supplement: Supplementary file 1 [file molecules-30-01453-s001.zip › molecules-3502327-supplementary.pdf]

S1:

Biological Assay:

3.2.1 Cell Culture

The human breast cancer cell line MCF-7 and ovarian cancer cell line SKOV-3 were obtained from an Egyptian company involved in the production of vaccines (VACSERA). All the following procedures were conducted in a sterile environment using Class II A2 Laminar flow biosafety cabinet. Cells were maintained in Dulbecco's modified Eagle's medium (DMEM) supplemented with 10% fetal bovine serum (FBS; Hyclone, UT), along with 100 U/mL penicillin G and 100 µg/ml. streptomycin. Culturing was carried out in a humid atmosphere with 5% CO_2 at 37°C. The cell culture media was obtained from Invitrogen-Life Technologies [66].

3.2.2 Cytotoxicity assay

Cell viability was assessed by the mitochondrial dependent reduction of yellow MTT (3-(4,5-dimethylthiazol-2-yl)-2,5-diphenyl tetrazolium bromide) to purple formazan[67]. MTT was purchased from Acros Organics™ Thermo Fisher Scientific, USA.

Cells were seeded at concentration of ($1x10^4$ cells/well) in fresh complete growth medium in 96-well plates and incubated at 37°C under 5% CO_2 for 24 h. The next day, cells was treated with tested drugs at five dose concentrations (500, 250, 125, 62.5, 31.25 ug/mL in serial dilutions) for 48 h. Additionally, 0.1% dimethyl sulfoxide (DMSO)-treated cells were used as negative control.

After treatment, the medium was aspirated, 20 µL of 5 mg/mL MTT reagent was added to each well and incubated for further four hours at 37 ºC under 5% CO_2. Next, the solution was removed and 100 µL of DMSO was added to the wells to dissolve the formed crystals. The resulted color, due to soluble formazan, was read at 570 nm with a microplate reader (800TSUV Biotek ELISA Reader, Agilent, Santa Clara, CA, USA), and corresponding optical densities were used for calculation of IC50 [68]. The results were analyzed as the percentage proliferation of the cells in respect to the concentration of the samples treated. A positive control which composed of 100 µg/mL doxorubicin was used as a known cytotoxic natural agent who gives 100% lethality under the same conditions. The inhibitory concentration (IC_{50}) was evaluated using Graph Pad prism software (San Diego, CA, USA) using the absorbance values and the following equation was used to determine the percentage of cell viability based on the absorbance readings of the test and control wells.

Viability = absorbance of drug / absorbance of control x 100

Cytotoxicity = 100- viability

3.2.3 Cell cycle and apoptosis detection by flow cytometry

Cell cycle assay was carried out using MCF-7 and SKOV-3 cell lines. Firstly, cells were seeded at a density of 3×10^5 cells/well for 24 hours. Then, cells were treated with IC50 doses of tested drugs based on the result of MTT assay (Table 1) and 0.1% DMSO treated cells were used as negative control. After 48 hours post-treatment, Cells were skinned gently and suspended in 50 µg/mL propidium iodide (PI) staining solution and 20 µg/mL RNase A and incubated for 1h. Cell cycle analysis was achieved using (Beckman Coulter Cytoflex, Indianapolis, Indiana, IN, USA) to identify cells in different cycle phases [69]Fluorescence was measured on flow cytometer and the obtained cell histograms were analyzed. Annexin V-FITC apoptosis Detection ELISA kit was used for analysis of apoptosis. The result was analyzed using Cell Quest 3.3 software. The experiment was performed in triplicates [70]

3.2.4 Gene expression analysis of Bcl-2 and Mcl-1 by RT-qPCR

MCF-7 and SKOV-3 cell lines were used for qPCR analysis of responded genes. The cells were seeded in 6-well plates at a density of 0.2×10^6 cells per well for 48 h after tested drugs treatment, compared to 0.1% DMSO negative control cells. Gene JET RNA Purification spin column Kit (K0731, Thermo Fisher Scientific, USA) was used for purification of RNA following the manufacturer's instructions. High-Capacity cDNA Reverse Transcription Kit (4368814, Thermo Fisher Scientific, USA) was used for cDNA synthesis. Quality and quantity of extracted RNA extraction and its cDNA copies were evaluated spectrophotometrically at 260 nm using the Nano Drop 2000C® (Thermo Fisher Scientific, Cairo, Egypt) and the absorbance ratio at 260/280 nm was used to verify RNA purity. Optimization for annealing temperatures of the primers, quantity of started cDNA and endogenous gene was performed before qPCR analysis with HERA SYBR® Green qPCR Kit system (Willowfort, Birmingham, UK).

Table 5 shows the sequences of the PCR primer pairs applied for each gene. All data were corrected to the endogenous control GAPDH.

Table 5: Bcl-2, Mcl-1 and GAPDH gene primer sequences

**Target**	***Sequence***
**Bcl-2**	*F 5'- ATCGCCCTGTGGATGACTGAGT-3'*<br>*R 5'- GCCAGGAGAAATCAAACAGAGGC-3'*
**Mcl-1**	*F 5'- CCAAGAAAGCTGCATCGAACCAT-3'*<br>*R 5'- CAGCACATTCCTGATGCCACCT-3'*
**GAPDH**	*F 5'- GTCTCCTCTGACTTCAACAGCG-3'*<br>*R 5'- ACCACCCTGTTGCTGTAGCCAA-3'*

Table S1:

SKOV cell line

Compound 2

**Tested compound**	**2**				
	**Different dilution (µg/ml)**				
	**500**	**250**	**125**	**62.5**	**31.25**
**Viability %**	**25.5±0.29**	**30.9±0.22**	**28.2±0.16**	**64.5±0.09**	**69.6±0.3**
**Cytotoxicity%**	**74.5±0.29**	**69.1±0.22**	**71.8±0.16**	**35.5±0.09**	**30.4±0.3**
***IC_{50} µg/ml**	**78.79**				

Compound 4a

**Tested compound**	**4a**				
	**Different dilution (µg/ml)**				
	**500**	**250**	**125**	**62.5**	**31.25**
**Viability %**	**21.7±0.19**	**25.2±0.14**	**35.4±0.08**	**54.4±0.14**	**63.2±0.11**
**Cytotoxicity%**	**78.3±0.19**	**74.8±0.14**	**64.6±0.08**	**45.6±0.14**	**36.8±0.11**
***IC_{50} µg/ml**	**96.78**				

Compound 4b

Tested compound	4b				
	Different dilution (µg/ml)				
	500	250	125	62.5	31.25
Viability %	30.1±0.64	36.9±0.18	33.8±0.16	53.8±0.24	59.1±0.21
Cytotoxicity%	69.9±0.64	63.1±0.18	66.2±0.16	46.2±0.24	40.9±0.21
*IC_{50} µg/ml	76.19				

Compound 5

Tested compound	5				
	Different dilution (µg/ml)				
	500	250	125	62.5	31.25
Viability %	42.5±0.11	48.9±0.12	49.2±0.15	73.4±0.24	77.1±0.22
Cytotoxicity%	57.5±0.11	51.1±0.12	50.8±0.15	26.6±0.24	22.9±0.22
*IC_{50} µg/ml	90.83				

MCF-7 Cell Line:

Compound 2

Tested compound	2				
	Different dilution (µg/ml)				
	500	250	125	62.5	31.25
Viability %	21.6±0.81	58.37±2.56	63.3±1.63	70.87±1.46	73.5±2.82
Cytotoxicity %	78.4±0.81	41.63±2.56	36.7±1.63	29.13±1.46	26.5±2.82
*IC_{50} µg/ml	260.97				

Compound 4c

Tested compound	4c				
	Different dilution (µg/ml)				
	500	250	125	62.5	31.25
Viability %	55±0.95	68.34±2.9	76±2.8	80.6±0.68	81.87±0.82
Cytotoxicity %	45±0.95	31.66±2.9	24±2.8	19.4±0.68	18.13±0.82
*IC_{50} µg/ml	577.97				

S2: Docking Studies:

Molecular Operating Environment (MOE) software was used to dock our most active derivatives (**2** and **4c**) into the 3D forms of both Bcl-2 and Mcl-1 with the proper ligands: The crystal structures of Bcl-2 (PDB:4LVT) at 2.6 Å resolution; and the crystal structure of Mcl-1 (PDB code: 6QYO)) at 2.6 Å resolution. As a representation of the conformational configurations with the most favorable binding energy, the predicted binding of the target derivatives to each Bcl-2 and Mcl-1 active pocket was found to be the best categorized scoring function. (ΔE).

S3: Spectroscopic analysis

C13 of the Most important Compounds

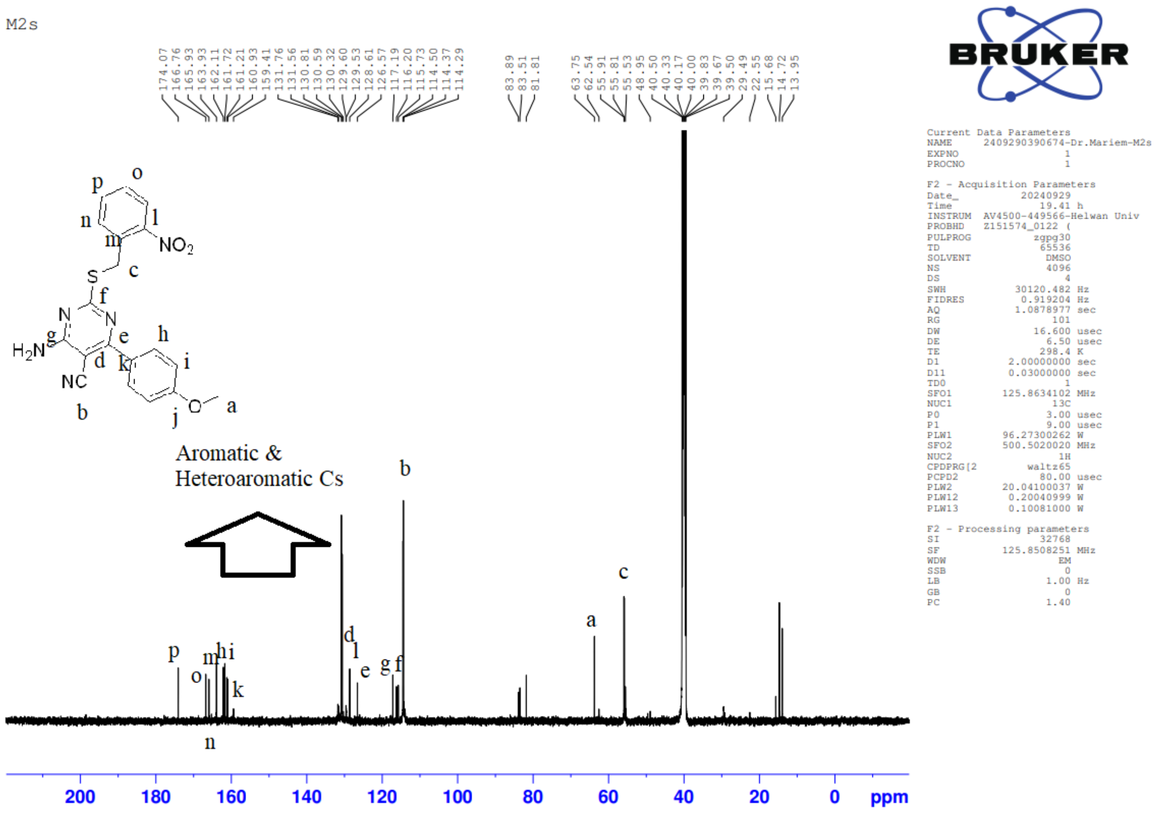

FigureS1a : C13 of Compound 2

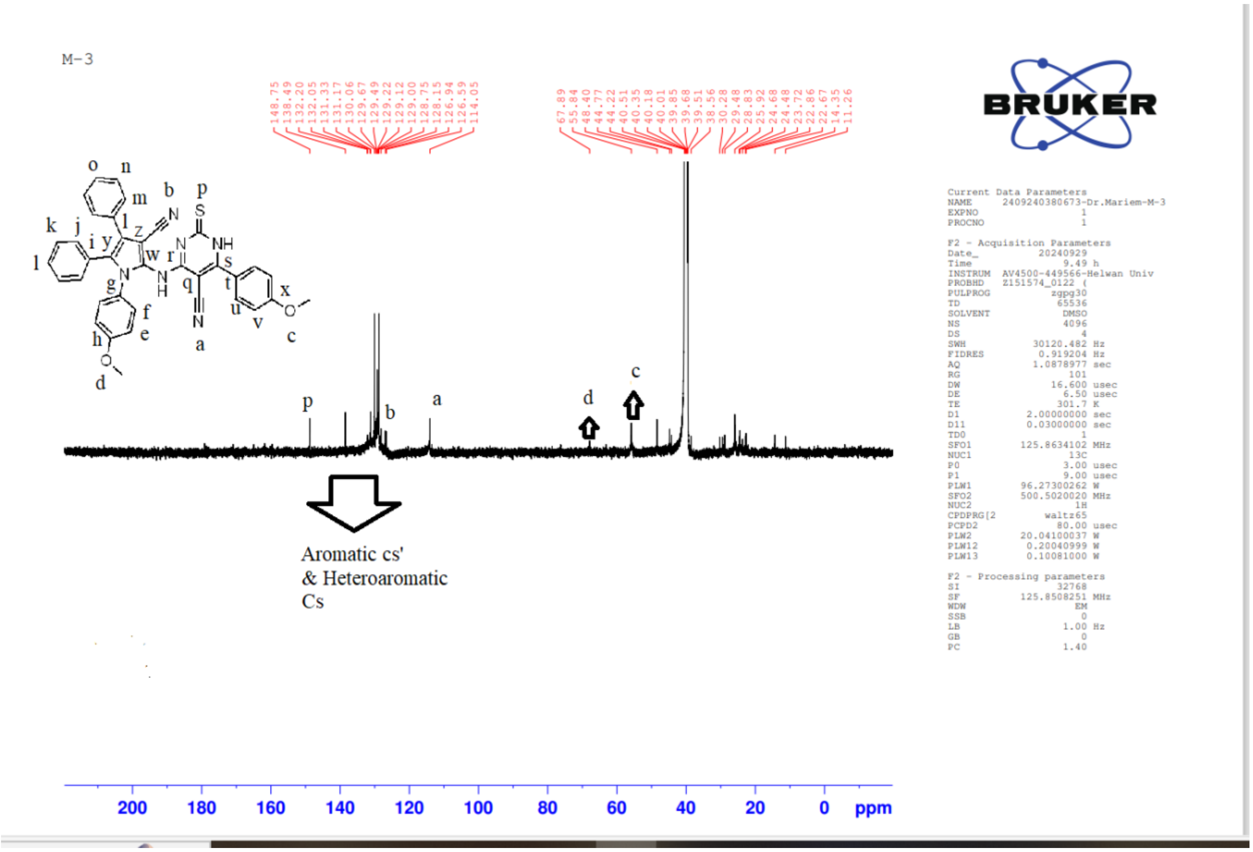

Figure S1b: C13 of Compound (4b)

Compound (2)

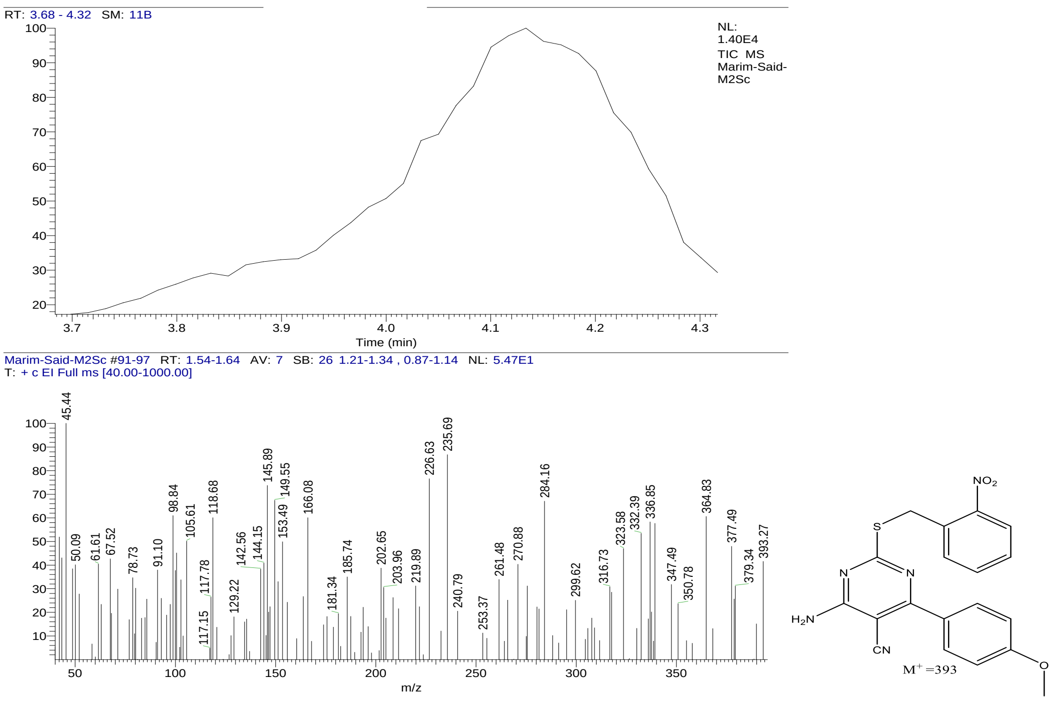

Figure S1c : Mass spectrum of compound (2)

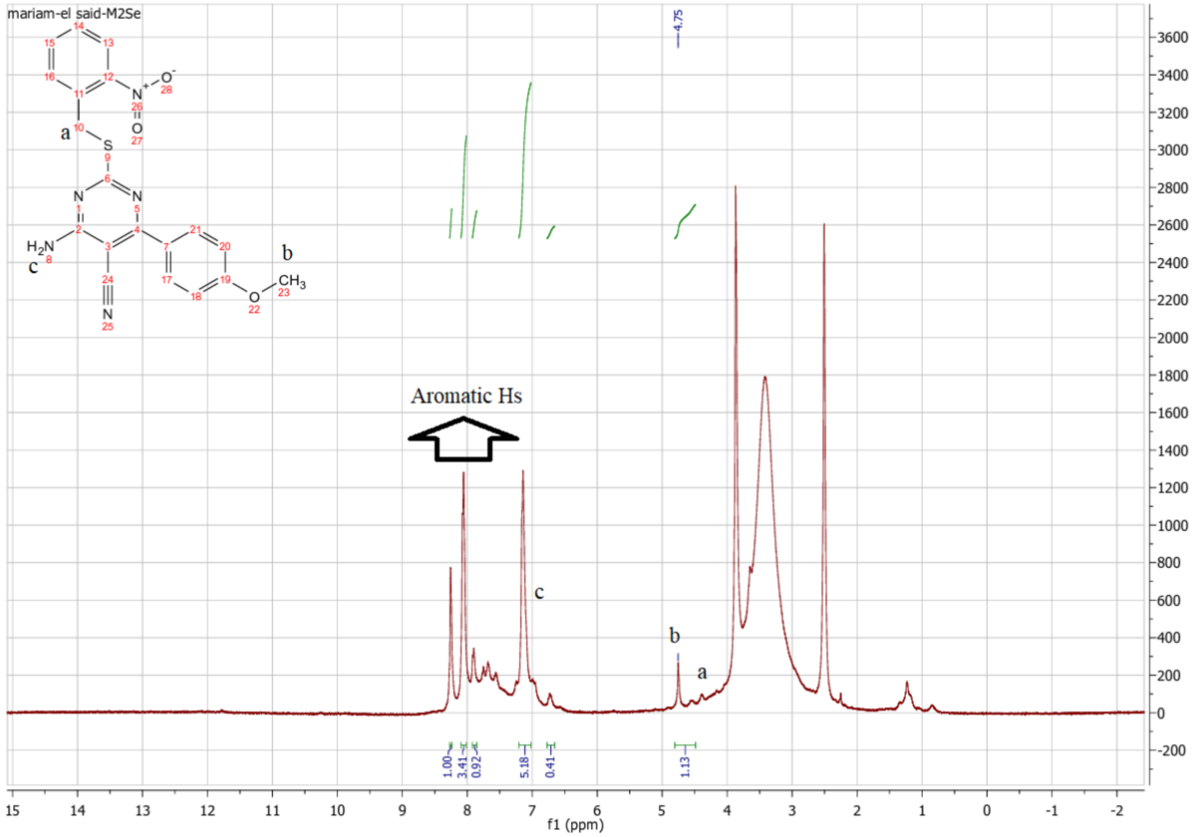

Figure S1d : ^{1}H-NMR spectrum of compound 2 DMSO-*d*$_6$.

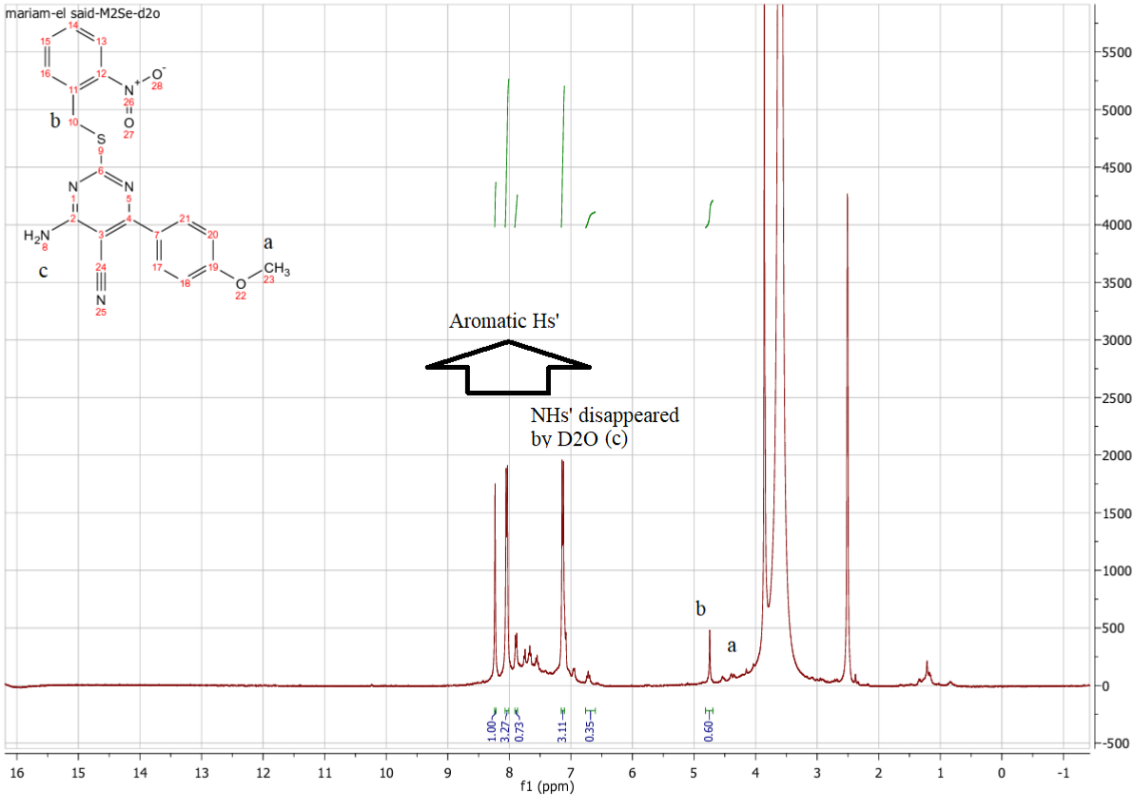

Figure S1e : ^{1}H-NMR spectrum of compound 2 D_2O.

Compound (4a)

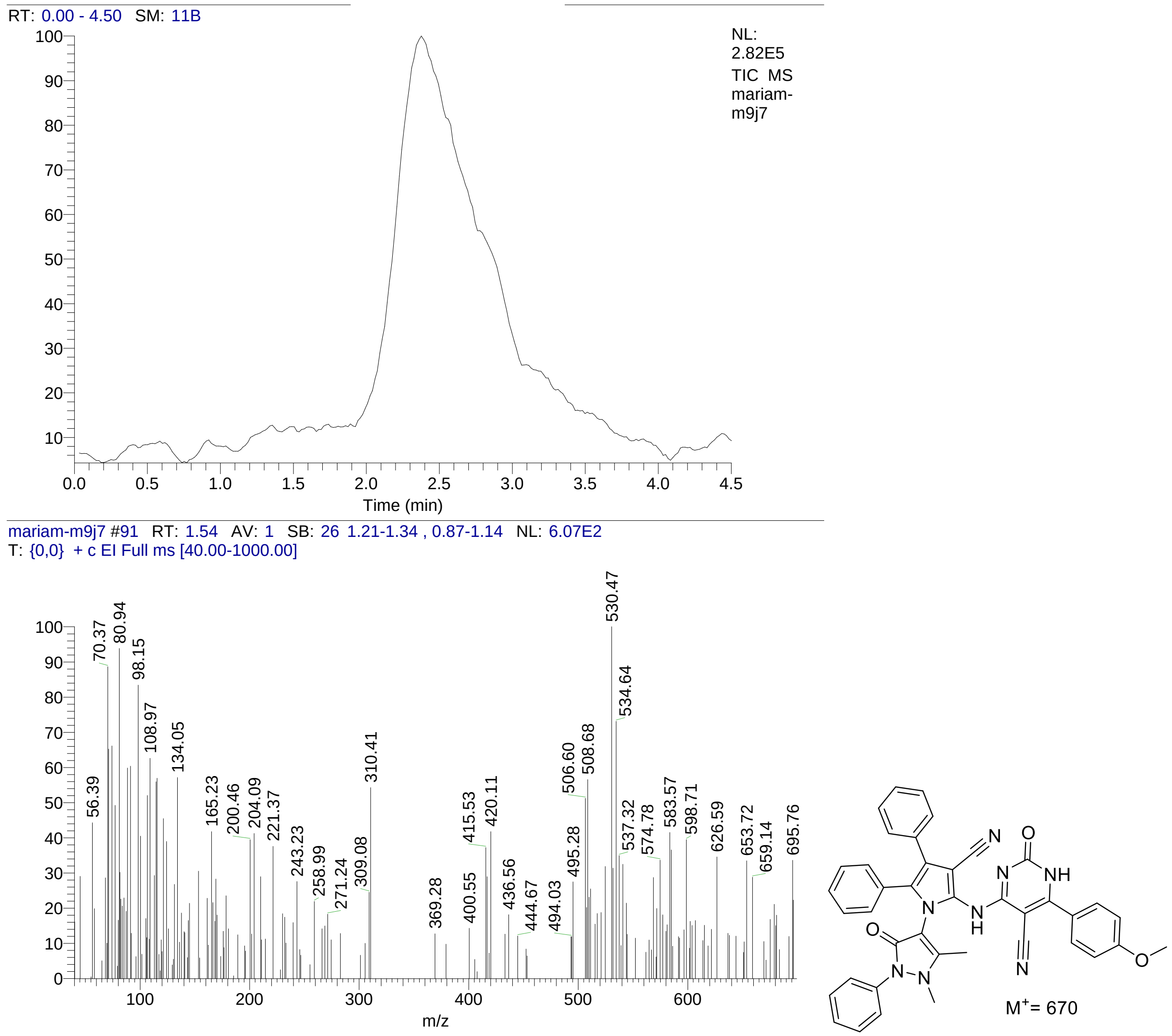

Figure S1f : Mass spectrum of compound (4a)

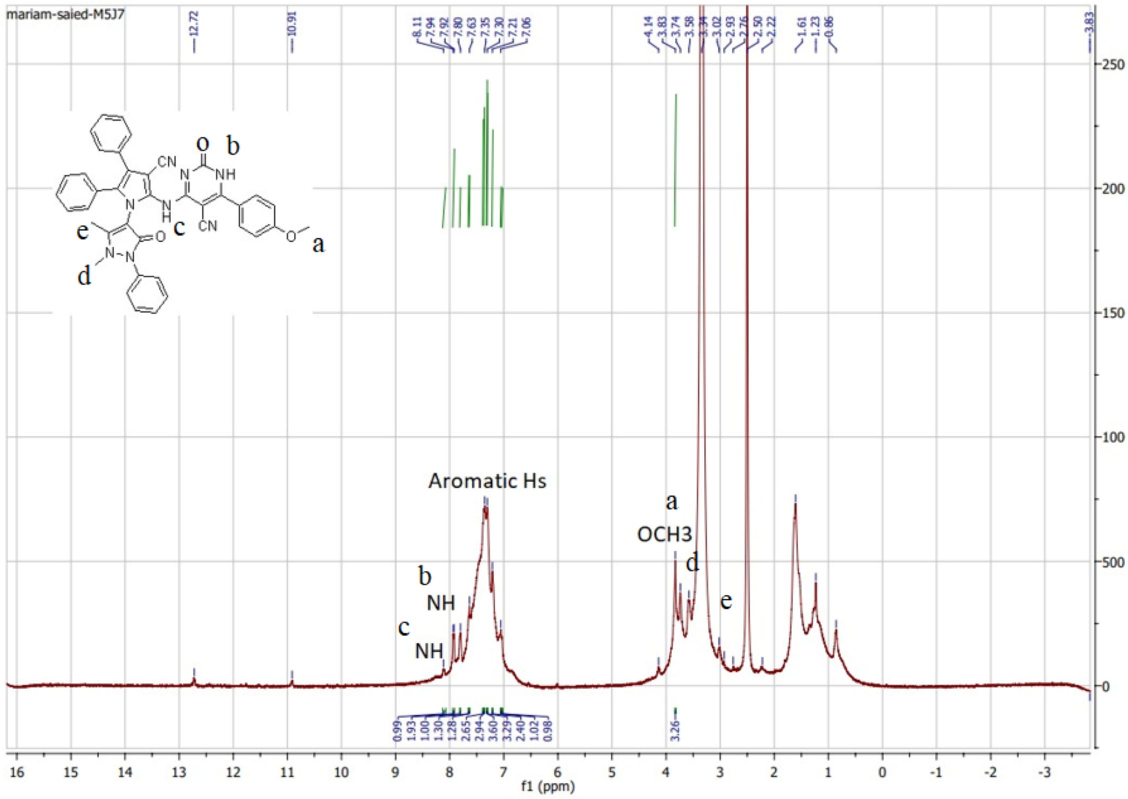

Figure S1g: ^{1}H-NMR spectrum of compound 4a DMSO-d_6.

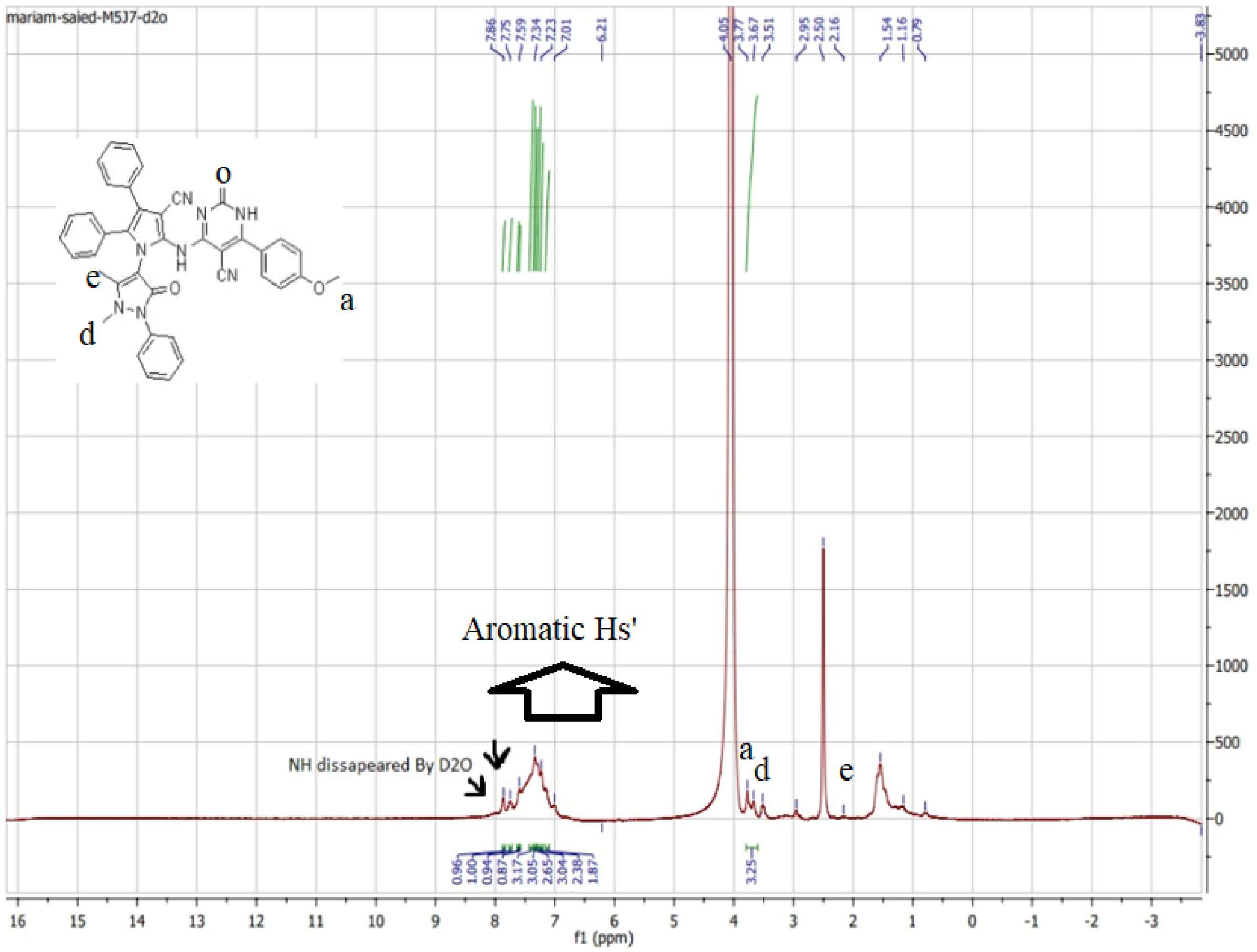

Figure S1h : ¹H-NMR spectrum of compound 4a D₂O

Compound (4b)

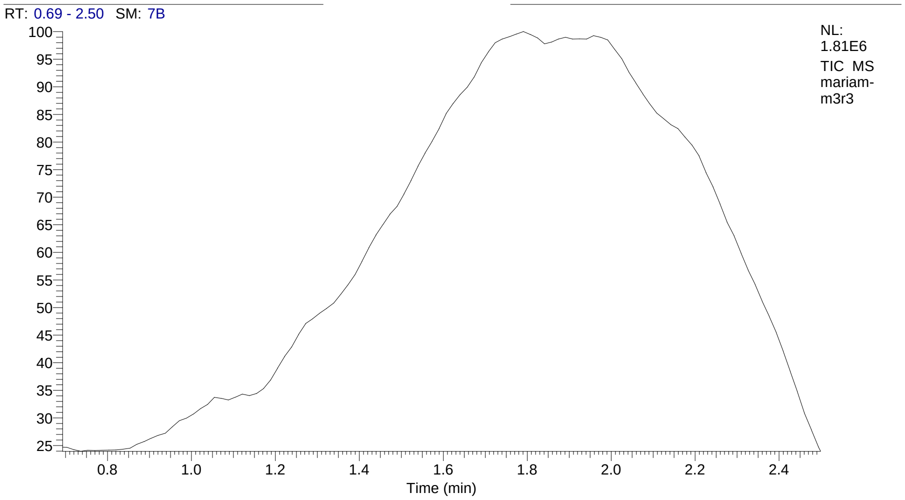

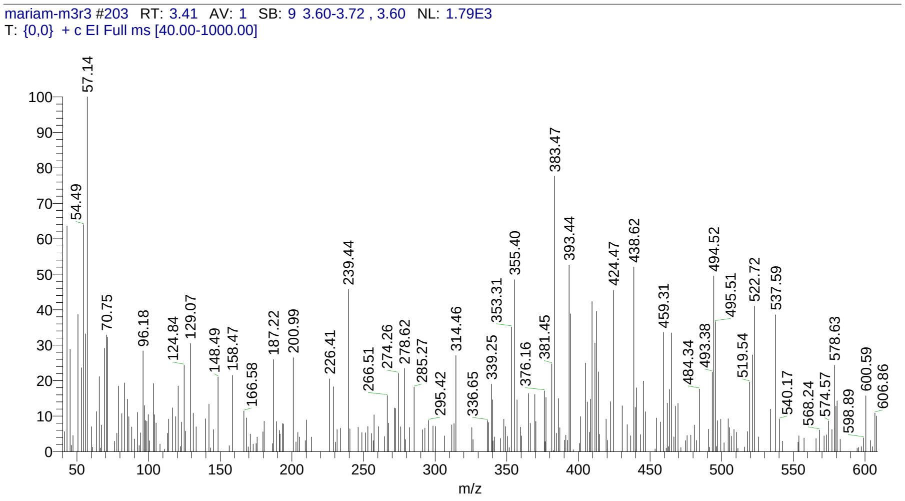

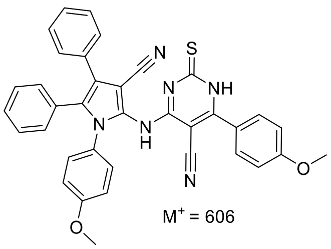

Mwt=606

Figure S1i : Mass Spectrum of compound 4b

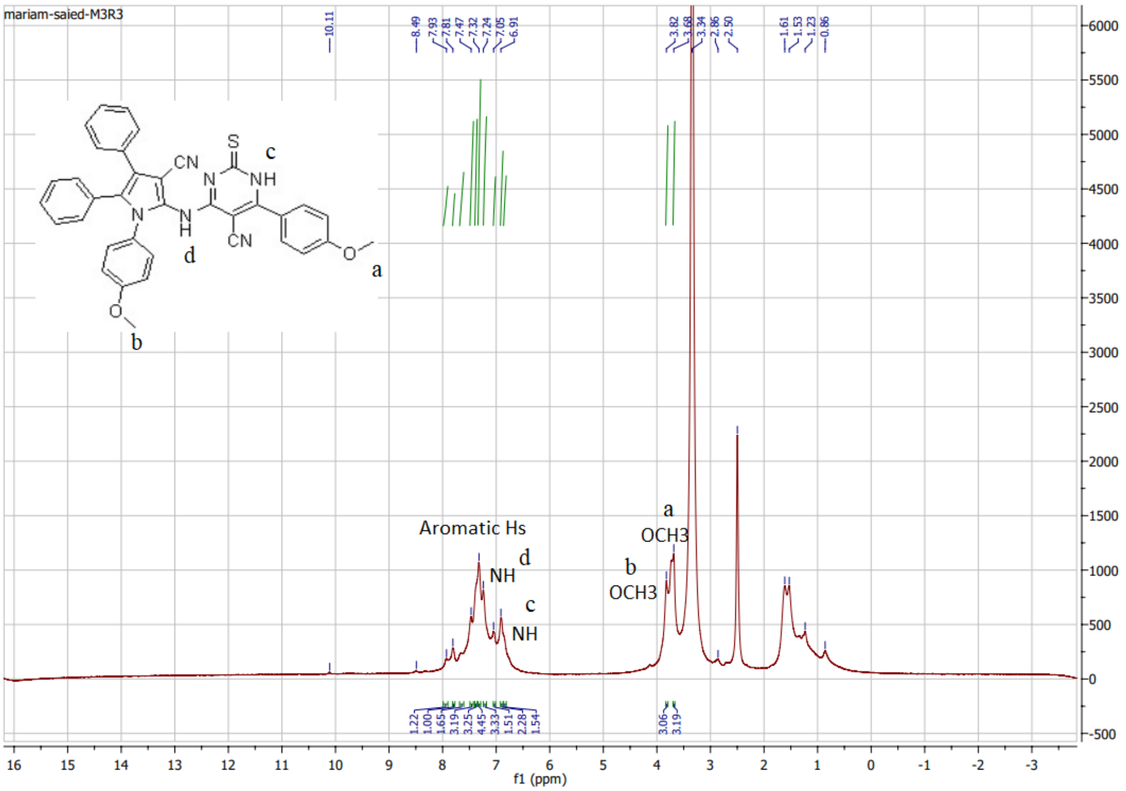

Figure S1j : ^{1}H-NMR spectrum of compound 4b

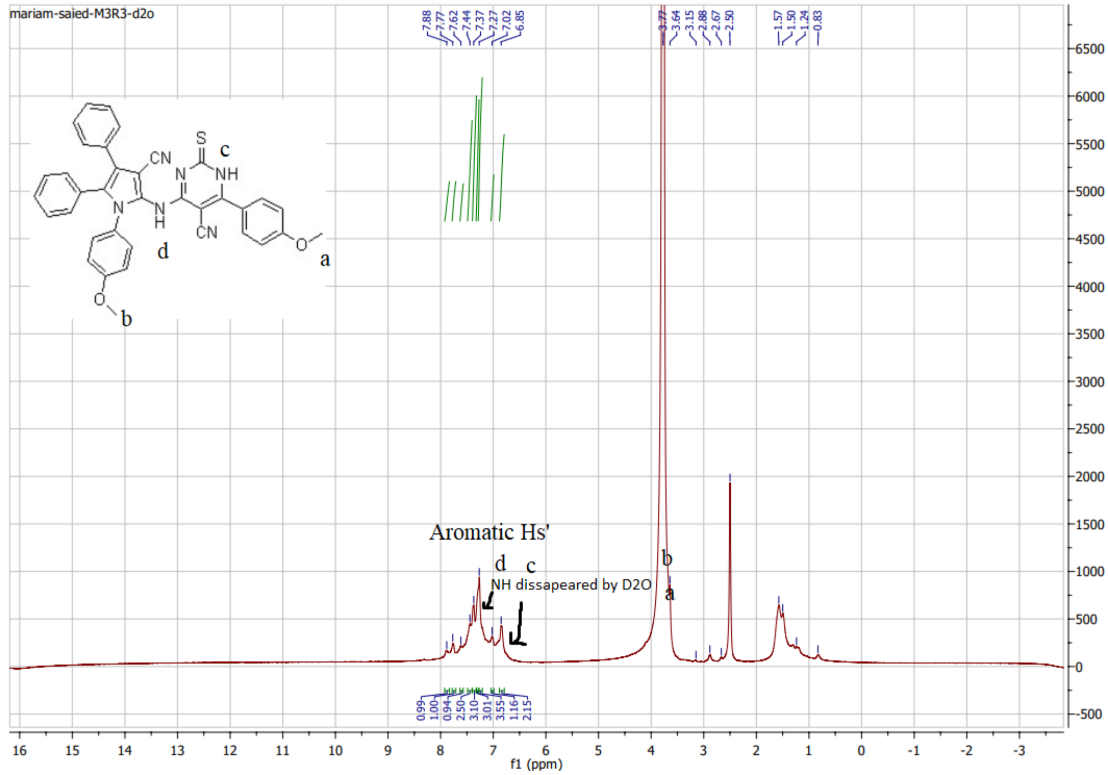

Figure S1k : ^{1}H-NMR spectrum of compound 4b D_2O.

Compound (4c)

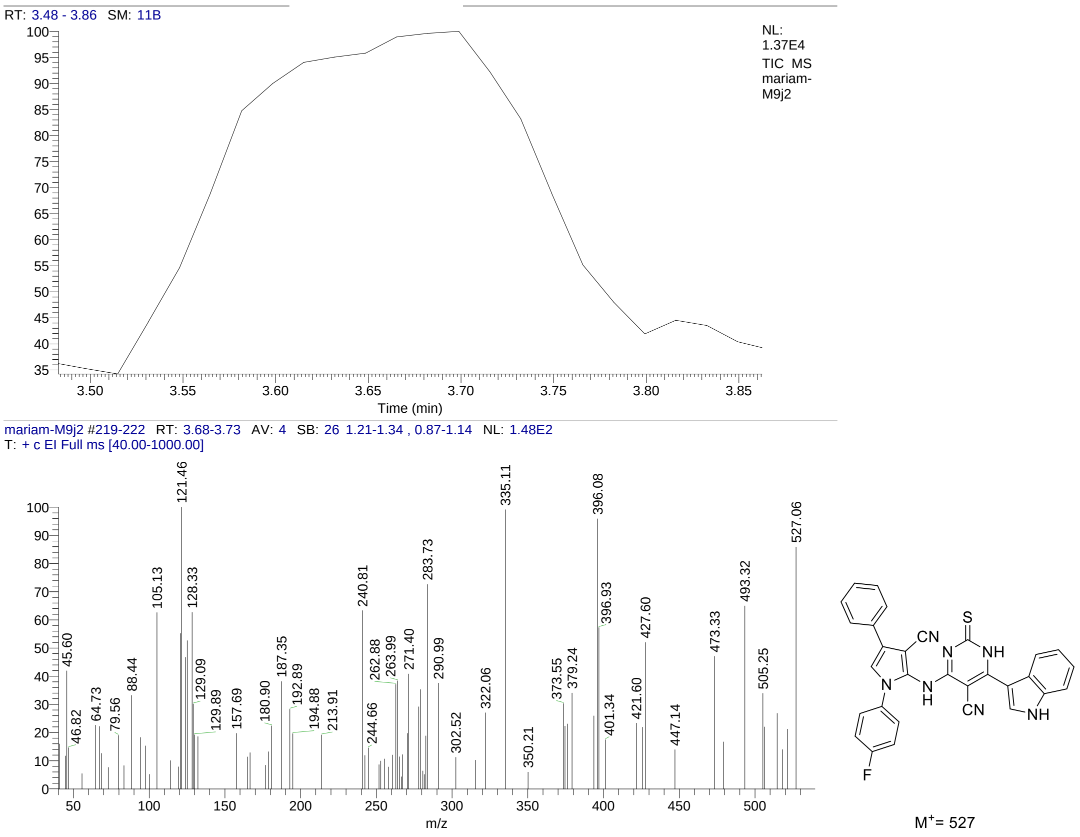

Figure S1L : Mass Spectrum of Compound 4c

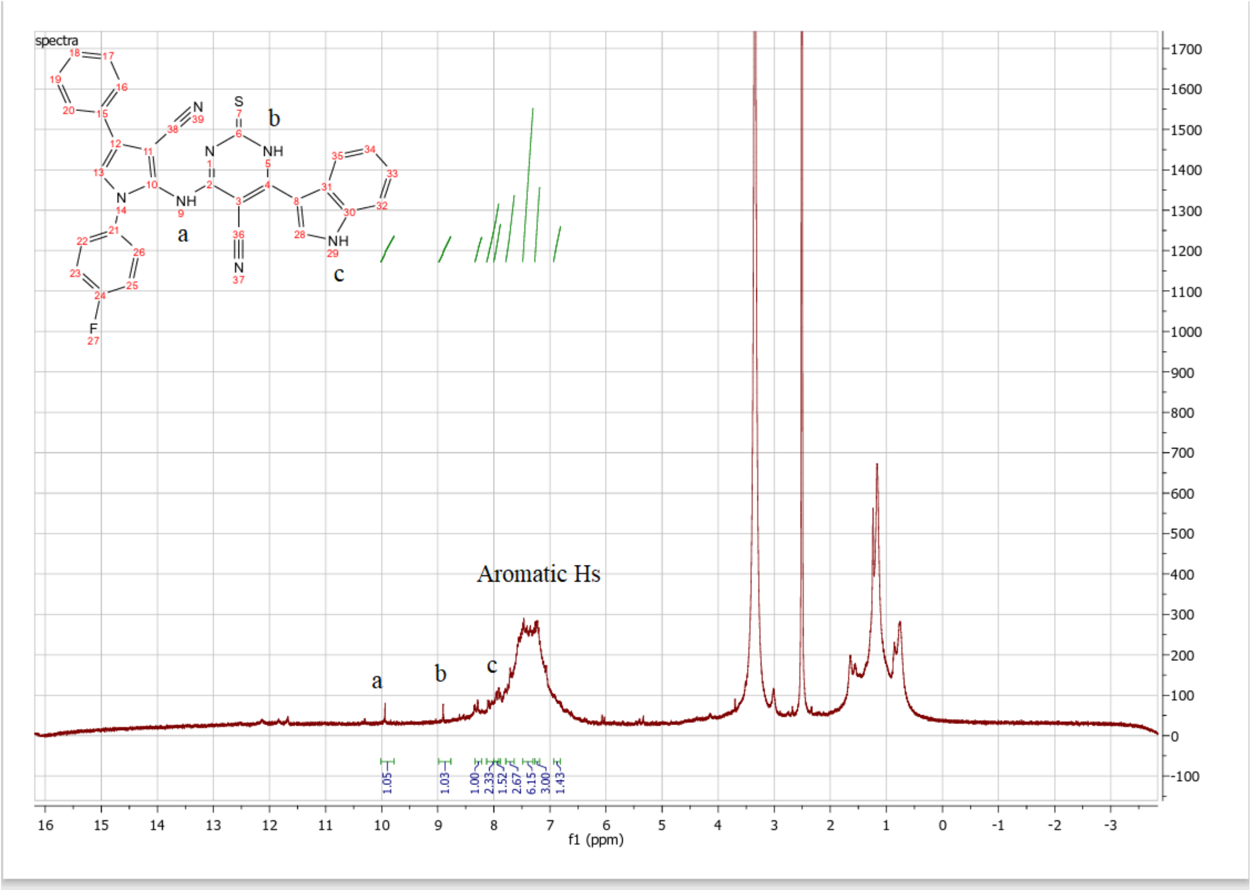

Figure S1m : ^{1}H-NMR spectrum of compound 4c DMSO-*d6.*

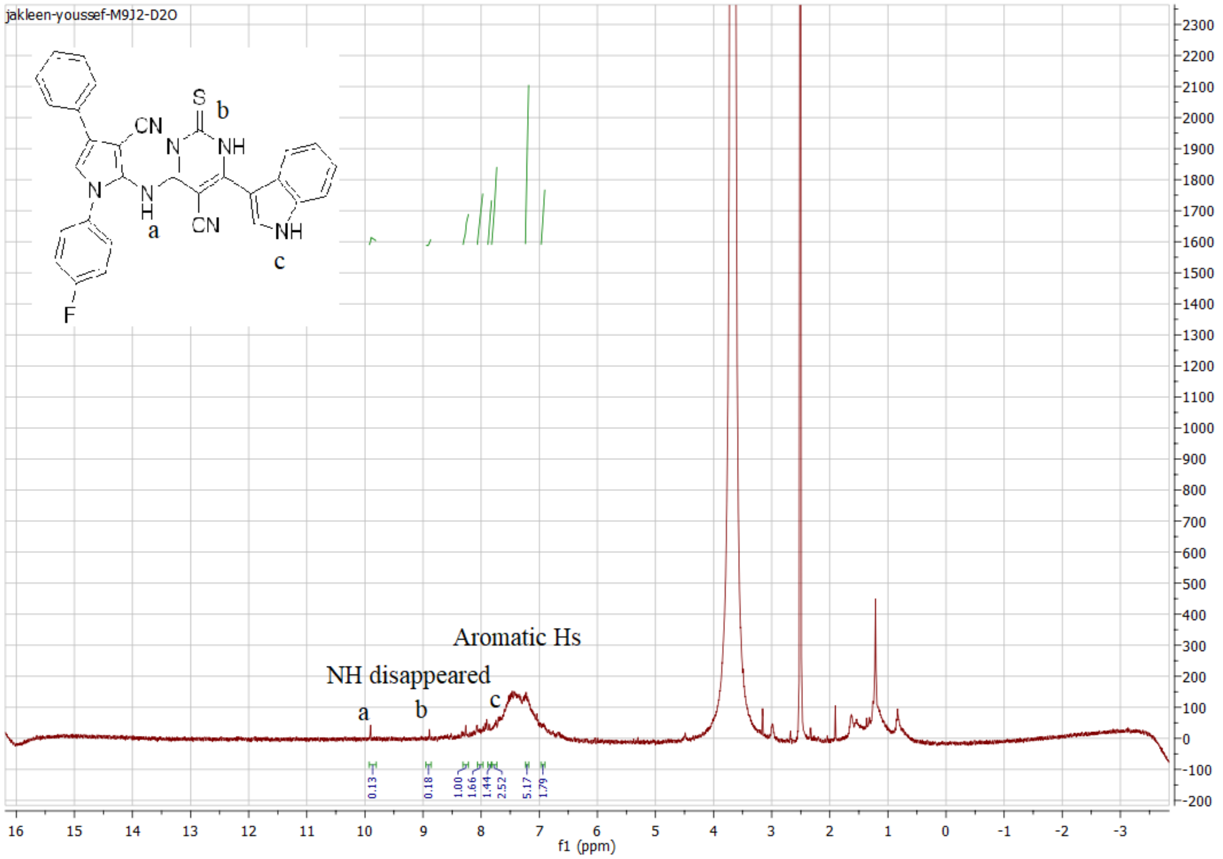

Figure S1n : ^{1}H-NMR spectrum of compound 4c D_2O.

Compound (5)

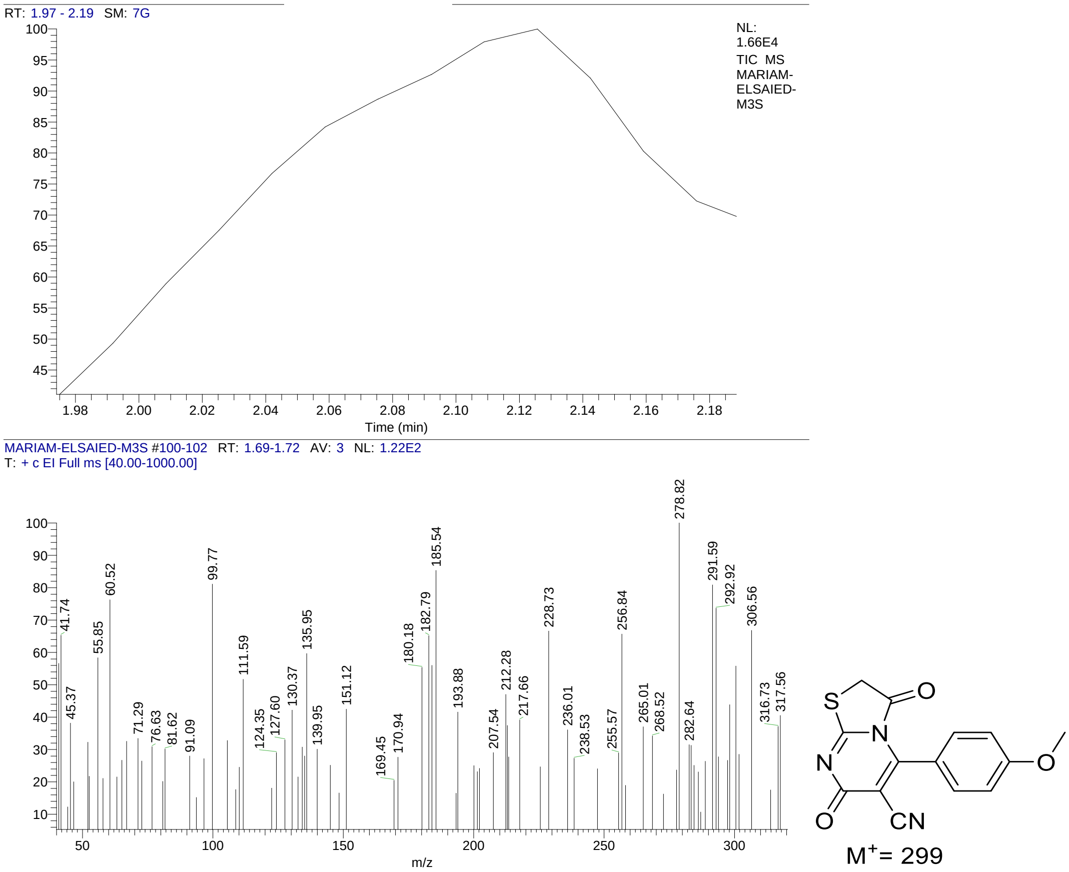

Mwt = 299

Figure S1o : Mass spectrum of compound 5

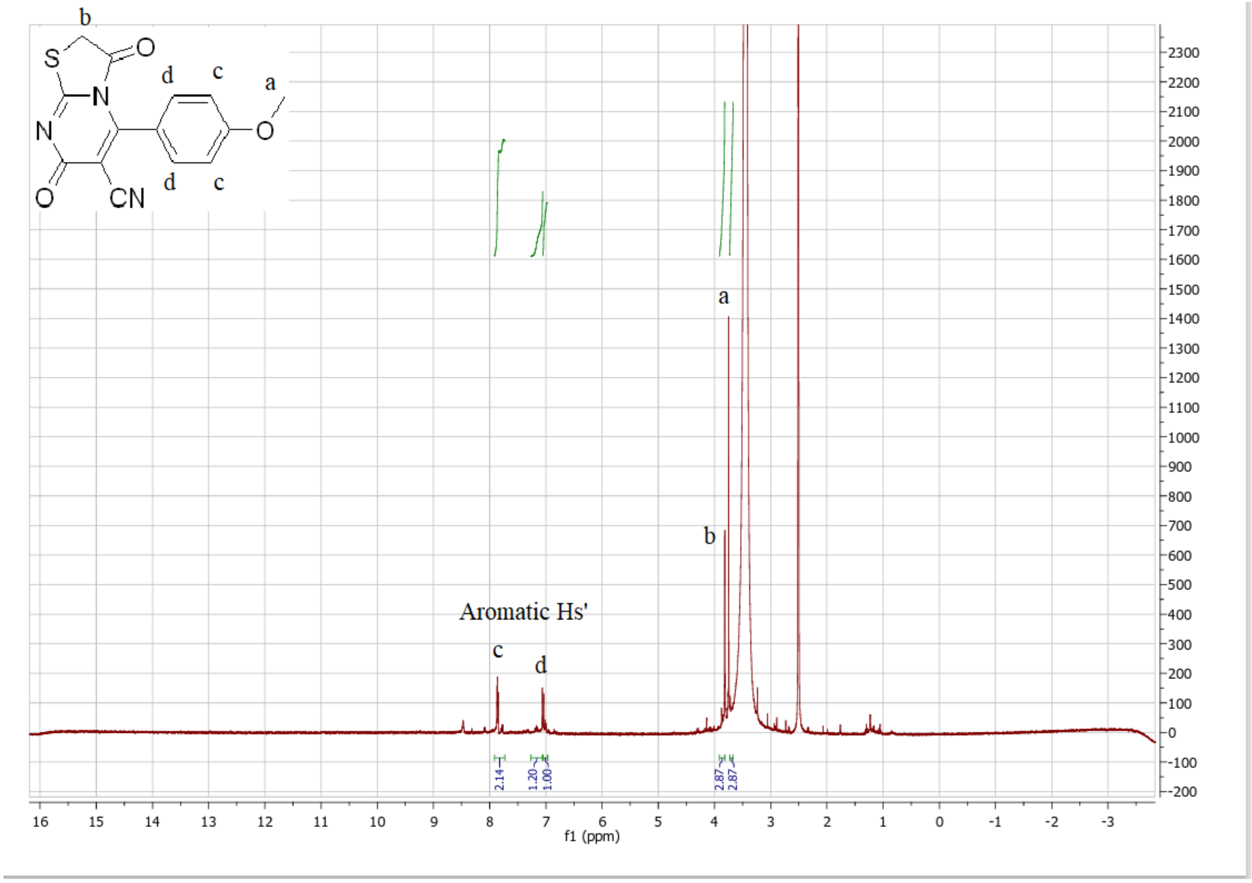

Figure S1p : ^{1}H-NMR spectrum of compound 5 DMSO-*d*6.
